# Identifying locations of re-entrant drivers from patient-specific distribution of fibrosis in the left atrium

**DOI:** 10.1371/journal.pcbi.1008086

**Published:** 2020-09-23

**Authors:** Aditi Roy, Marta Varela, Henry Chubb, Robert MacLeod, Jules C. Hancox, Tobias Schaeffter, Oleg Aslanidi

**Affiliations:** 1 Department of Biomedical Engineering, School of Biomedical Engineering & Imaging Sciences, King’s College London, St Thomas’ Hospital, London, United Kingdom; 2 National Heart and Lung Institute, Imperial College London, London, United Kingdom; 3 Cardiothoracic Surgery, Stanford University, United States of America; 4 Bioengineering Department, University of Utah, Salt Lake City, Utah, United States of America; 5 School of Physiology and Pharmacology, Cardiovascular Research Laboratories, University of Bristol, Bristol, United Kingdom; 6 Physikalisch-Technische Bundesanstalt, Berlin, Germany; Universiteit Gent, BELGIUM

## Abstract

Clinical evidence suggests a link between fibrosis in the left atrium (LA) and atrial fibrillation (AF), the most common sustained arrhythmia. Image-derived fibrosis is increasingly used for patient stratification and therapy guidance. However, locations of re-entrant drivers (RDs) sustaining AF are unknown and therapy success rates remain suboptimal. This study used image-derived LA models to explore the dynamics of RD stabilization in fibrotic regions and generate maps of RD locations. LA models with patient-specific geometry and fibrosis distribution were derived from late gadolinium enhanced magnetic resonance imaging of 6 AF patients. In each model, RDs were initiated at multiple locations, and their trajectories were tracked and overlaid on the LA fibrosis distributions to identify the most likely regions where the RDs stabilized. The simulations showed that the RD dynamics were strongly influenced by the amount and spatial distribution of fibrosis. In patients with fibrosis burden greater than 25%, RDs anchored to specific locations near large fibrotic patches. In patients with fibrosis burden below 25%, RDs either moved near small fibrotic patches or anchored to anatomical features. The patient-specific maps of RD locations showed that areas that harboured the RDs were much smaller than the entire fibrotic areas, indicating potential targets for ablation therapy. Ablating the predicted locations and connecting them to the existing pulmonary vein ablation lesions was the most effective in-silico ablation strategy.

## Author summary

Atrial fibrillation (AF) is the most common cardiac arrhythmia and a huge healthcare problem, but its mechanisms are incompletely understood, and clinical therapies such as catheter ablation (CA) have poor long-term outcomes. This is due to the empirical nature of the procedure and lack of mechanistic knowledge of optimal ablation sites and strategies in patients whose atria is altered by AF-induced structural remodelling. In this study, we developed 3D atrial models with patient-specific geometry and distribution of fibrosis obtained from AF patient imaging and applied the models to explore the mechanisms of re-entrant drivers (RD) sustaining AF. Moreover, we used the novel mechanistic knowledge to simulate CA based on the model predictions and compared its success with existing clinical CA strategies. We discovered that the RD dynamics were strongly influenced by the spatial distribution of fibrosis, and RDs typically anchored to specific locations near large fibrotic patches. Virtual ablations of such anchoring locations by connecting them with linear lesions to the nearest pulmonary veins (PV) had superior efficacy compared to clinically used strategies such as the PV isolation. After incorporating further patient-specific information and careful validation, the proposed in-silico approach can help evaluate and potentially guide CA therapy in the clinic.

## Introduction

The prevalence of atrial fibrillation (AF) is increasing to epidemic proportions: worldwide over 33 million individuals have AF [1]. Rhythm control strategies for maintaining sinus rhythm, such as antiarrhythmic drugs, can lead to significant improvements of cardiac output and quality of life. Over recent decades, catheter ablation (CA) therapy has also become a first-line treatment for AF. Radiofrequency CA is aimed at destroying arrhythmogenic tissue areas in the atria via high energy delivery through a catheter, and it is the only treatment with a proven long-term curative effect [2]. However, treatments of AF are complicated by its mechanisms for self-sustenance, such as the presence of AF-induced electrical and structural remodelling that generates more treatment-resistant arrhythmia [3,4]. Therefore, even advanced CA procedures have suboptimal long-term outcomes in patients with chronic forms of AF: over half of the patients return for additional treatment within three years [5]. This can be explained by the highly empirical nature of CA therapy, which targets “usual suspect” areas without knowledge of the underlying arrhythmogenic mechanisms. Thus, CA therapy based on electrical isolation of the pulmonary veins (PV) has low success rates in chronic AF patients, where extensive ablation of remodelled non-PV areas is commonly applied [6].

AF has been strongly linked with structural changes of the atria, especially with the development of atrial fibrosis identified from medical imaging [7]. Mechanistically, fibrosis is a product of structural remodelling of atrial tissue, which results in the deposition of a collagenous matrix in response to mechanical stress on the atria during AF. Since collagen is non-conductive, it can slow down or completely block propagation of electrical excitation waves, thus providing a substrate for AF [8]. Since the progression of AF has been linked with high levels of atrial fibrosis, the quantification of fibrosis from late-gadolinium enhanced magnetic resonance imaging (LGE MRI) has been applied for the stratification of AF patients, and a higher fibrotic burden has been associated with more severe AF and reduced success rate of CA procedures [9,10]. Moreover, areas of patchy fibrosis in the atria show high levels of arrhythmogenic electrical activity and ablation around such patient-specific areas can improve therapy success [11]. Recent clinical studies have reported that low-voltage areas, identified from atrial tissue mapping and associated with the presence of fibrosis, can be directly targeted by CA to improve patient outcomes [12,13]. However, such areas can be quite large, and their ablation can result in substantial damage of the atria, impairing its function.

Thus, image-guided CA procedures are increasingly used to move away from empirical therapy and improve the patient outcomes. However, even advanced imaging systems do not provide crucial functional information about the origins of arrhythmogenesis, and the success of image-based patient stratification and CA guidance remains suboptimal. Image-based computational modelling can provide such information by predictive simulations of 3D atrial function in a given patient, particularly by linking their distribution of fibrosis with AF arrhythmogenesis.

Recent computational studies of patient-specific atrial models, based on the reconstruction of fibrosis from LGE MRI, have provided first insights into for the role of fibrosis in the dynamics of electrical re-entrant drivers (RDs) sustaining AF. Thus, McDowell et al. [14] showed that patient-specific distribution of fibrosis was a critical component of AF initiation and maintenance, with RDs only induced in atrial models with high level of patchy fibrosis. Moreover, patient-specific models demonstrated that AF was sustained by RDs persisting in fibrosis border zones characterized by specific regional fibrosis architecture metrics Zahid et al. [15]. Recent work from our group provided mechanistic insights into these effects, demonstrating that RDs stabilize in border zones (BZ) of patchy fibrosis, where slow electrical conduction facilitated the development of re-entrant circuits within relatively small regions [16,17]. These computational model predictions have been validated by a recent clinical study that linked the patient-specific LGE areas with locations of RDs recoded using electrocardiography [18]. Moreover, recent computational modelling studies by Boyle et al. [19,20] have illustrated correlations between the locations of RDs predicted by patient-specific atrial models and the respective locations found with electrocardiographic imaging (ECGI) and focal impulse rotor modulation (FIRM). In addition to shedding light into the role of fibrosis in the RD dynamics, these studies pave the way to the identification of patient-specific RDs locations from image-based 3D atrial models.

In this study, patient-specific atrial models were applied to explore links between MRI-derived fibrosis distributions and RD locations. Specifically, the aims of this study were to 1) apply 3D left atrial (LA) models based on LGE MRI to explore the dynamics of RD stabilization in patient-specific fibrotic areas, 2) generated personalized RD location maps–potential ablation targets–that identify the regions with the highest probability of RDs anchoring. The maps were correlated with the image-based Utah fibrosis score [7]. The first aim can help clarify what characteristics of fibrotic distributions affect the RD dynamics, while the second may in a longer term predict patient-specific CA targets that do not require extensive atrial voltage mapping and ablation in a patient, and hence can facilitate faster and more efficient therapy. Finally, we simulated virtual CA of the targets identified in the image-based LA models in a subset of AF patients and compared these to clinical CA strategies.

## Methods

**Fig 1:**
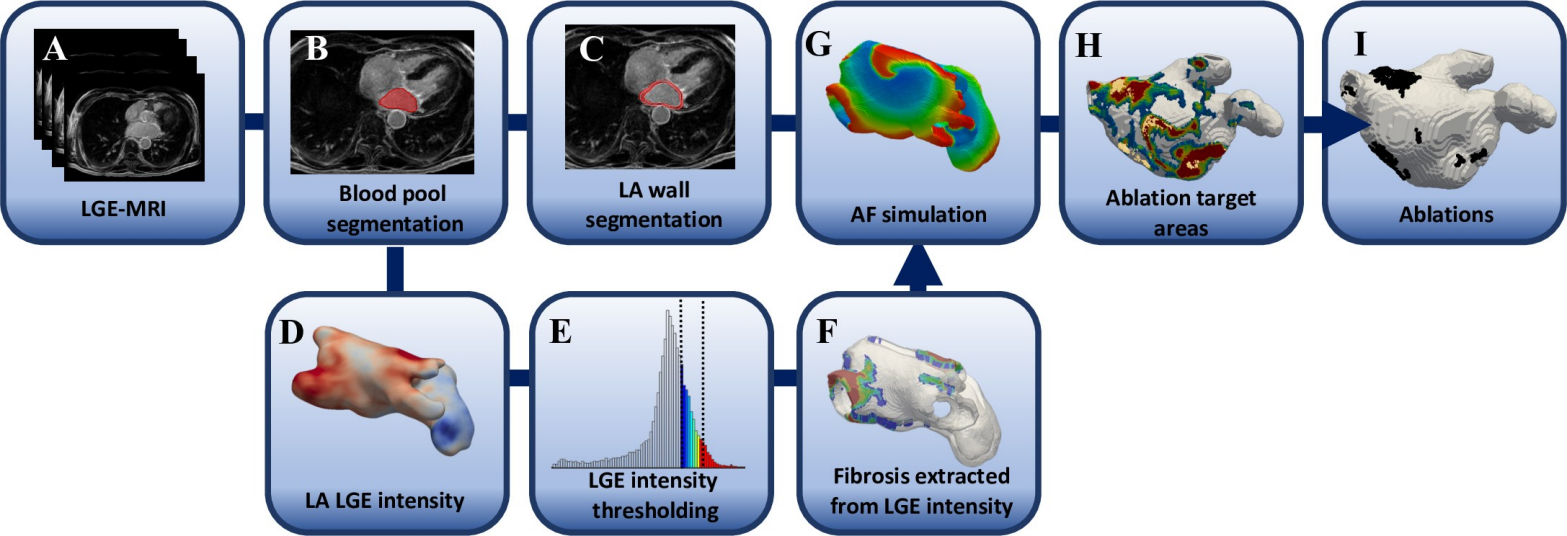
Workflow for identifying patient-specific target areas with the highest probability of harbouring RDs. The personalized left atrial models are generated by segmentation of patient-specific LA geometry and fibrotic regions from LGE-MRI scans.

The study applied fibrosis distributions derived from patient LGE MRI data to build realistic 3D LA models and simulate the patient-specific RD dynamics. The models were generated using the general image-based computational workflow illustrated in **Fig 1**.

### Atrial electrophysiology model

All simulations were performed by solving the monodomain equation with the Fenton-Karma model [21] modified to accurately describe basic electrophysiological properties of AF-remodelled atrial cells [22]. This atrial Fenton-Karma (aFK) cell model, despite being simple and phenomenological, accurately captures the main characteristics of atrial action potential and its restitution properties. The simplification enables keeping computational time to be kept relatively short, which is crucial for large-scale 3D atrial simulations. A finite-difference PDE solver based on central finite differences and explicit Euler schemes were used with spatial and temporal resolutions of 0.3 mm and 0.005 ms, respectively, as described in previous modelling studies [23,24]. The computer code implementing the PDE solver was parallelized under MPI and run on a 64-core local HPC server: simulations of 1 s of activity in the left atrium took approximately 1 hour. Our model was isotropic and the diffusion tensor was replaced by a scalar diffusion coefficient *D* of 0.1 mm^2^ ms^-1^, chosen to match an atrial conduction velocity of 0.6 ms^-1^ typical of AF [25].

### 3D patient-specific LA models

Briefly, the patient specific LA geometries and fibrosis distribution were reconstructed from LGE-MRI to generate patient-specific LA models. RDs were initiated at multiple (8–12) locations in each LA model using a cross field protocol and their tip was tracked for 6s. The location where each RD stabilised after 6s was identified and labelled as being part of: (i) healthy tissue, (ii) PV region or (iii) fibrotic patches. Each tissue voxel was additionally assigned a probability value, by investigating how many times it was visited by an RD tip over the course of the simulation. The most likely locations of RDs are expected to be prime targets for CA and were thus defined as the target areas (TA) (see workflow in **Fig 1**). Note that the RDs which anchored to the PV openings, which resulted from clipping of the PVs near their ostia and were non-physiological, were excluded from the definition of the TAs. Finally, virtual CA was performed on the identified TA regions and compared to existing clinical strategies.

### Imaging of patient-specific LA geometries and fibrosis distribution

Two persistent AF (PsAF) and four paroxysmal AF (PAF) patients (see Table 1) were imaged under ethical approval and following written informed consent as a part of the study by Chubb et al. [26]. All imaging was performed on a 1.5T Phillips MRI scanner and included a LGE MRI sequence: a 3D inversion recovery spoiled gradient echo, acquired 20–30 min after the administration of the extra-cellular gadolinium-based contrast agent Gadovist (Bayer Healthcare Pharmaceuticals). These images were acquired using cardiac and respiratory gating, with a spatial resolution of 1.3 x 1.3 x 4 mm^3^. Further information about the used LGE sequence can be found in [26].

**Table 1:** Characteristics of the 6 AF patients whose LA models were used in the study.

Patient	AF	Age	Gender	% Fibrosis	Utah Score
P1	PsAF	71	Male	39	4
P2	PsAF	65	Male	29	3
P3	PAF	72	Male	25	3
P4	PAF	57	Male	22	3
P5	PAF	58	Male	16	2
P6	PAF	53	Male	11	2

PsAF: persistent AF, PAR: paroxysmal AF. The patients have been labelled 1 to 6 (column 1), in the decreasing order of their fibrosis burden (column 5) and assigned a Utah score.

The LA geometries were obtained by manual segmentation of the LGE MR images (**Fig** 1A) using MITK Workbench [27], where the endocardial wall was identified by segmenting the LA blood pool (**Fig 1**B). The epicardial wall was generated by dilating the endocardial wall by 3 mm (**Fig 1**C), which is reported as the average LA wall thickness in AF patients [28]. The PV sleeves were removed, and patient-specific LA geometries were synthesized with a resolution of 0.3 mm, to be used in finite difference simulations.

**Fig 2:**
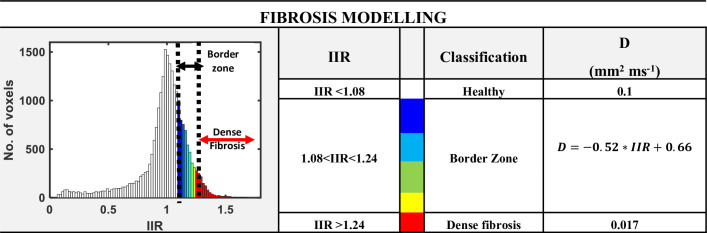
Segmentation and modelling of fibrosis. Labelling of voxels in the LA patient geometries according to their LGE MRI intensity ratio (IIR) relative to the blood pool. Voxels with IIR>1.24 are considered to be part of a fibrotic core. IIR<1.08 corresponds to healthy tissue and the intermediate values of IIR form a fibrotic border zone with intermediate properties.

**Fig 3:**
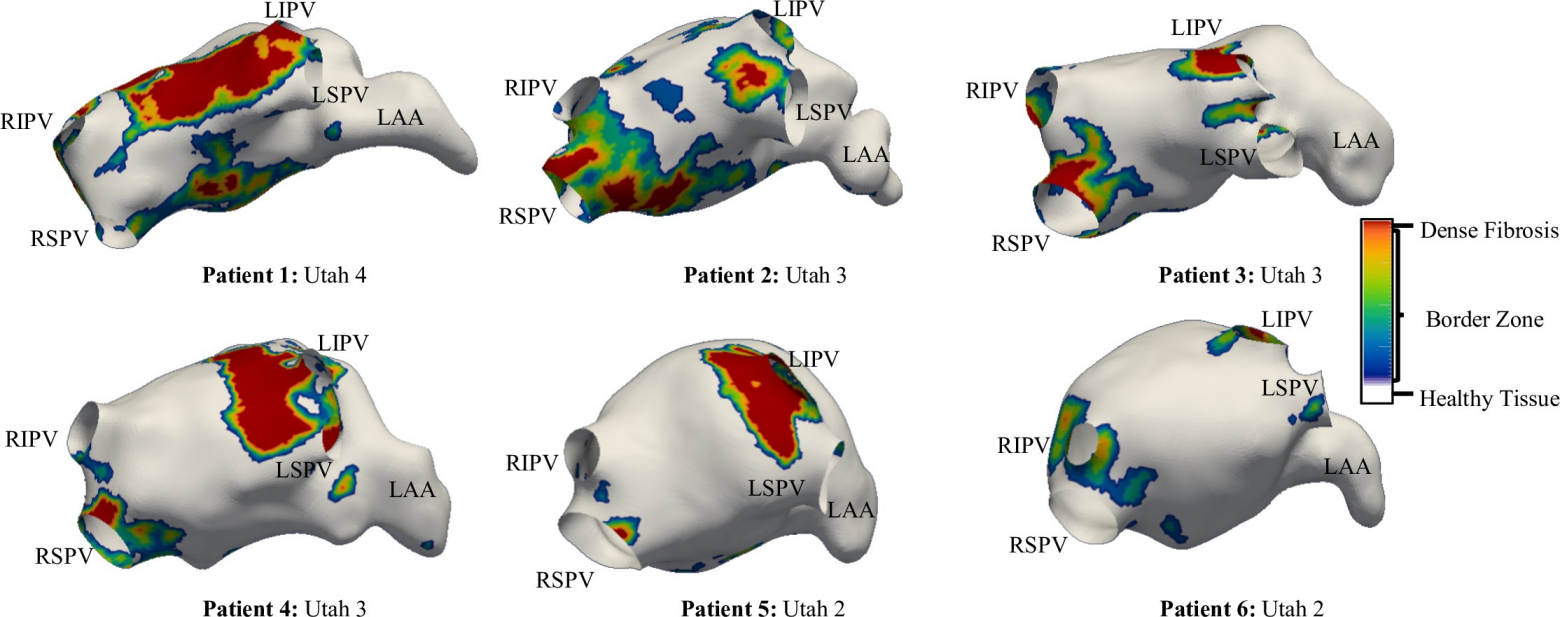
Patient-specific fibrosis distribution in 6 AF patients with Utah scores from 2 to 4. The fibrotic regions are colour-coded to show dense fibrotic tissue (red) surrounded by a BZ of intermediate properties. The healthy atrium is shown in white. LIPV: left Inferior PV, RIPV: Right Inferior PV, LSPV: left Superior PV, RSPV: Right Superior PV and LAA: Left Atrial Appendage.

The patient-specific fibrosis distribution in the LA of each AF patient was generated based on the image intensity of the LGE MRI data (**Fig 1**D–**1**F). A voxel was considered to be part of a fibrotic patch when the ratio of the voxel intensity to the mean blood pool intensity, the image intensity ratio (IIR), (**Fig 1**E) exceeded an empirical threshold [29,30]. Voxels were labelled as being healthy tissue (IIR < 1.08, white region of the histogram in **Fig 2**), dense fibrosis (IIR > 1.24, red region of the histogram) and the region around the dense fibrotic patch corresponds to the BZ (1.08 < IIR < 1.24, histogram with colours blue to yellow). The lower IIR threshold of 1.08 was obtained as an average of the previously proposed values of 1.2 [30] and 0.97 [29]. While, the upper IIR threshold limit of 1.24 was chosen for the models as all the LGEs were post ablation and therefore, the IIR threshold value reported for dense scar, 1.32 [30] which represents pre-exiting ablation lesions was reduced by 6%. The fibrosis maps obtained by intensity thresholding of LGE MRI data for all the 6 AF patients are depicted in **Fig 3**. These fibrotic regions were registered (Paraview, Kitware) and subsequently projected onto the LA geometry (Matlab, Mathworks Inc), such that the fibrosis patches were fully transmural (**Fig 1**H).

Each of 6 patients was classified into one of the following four groups [31] depending on their relative fibrosis burden (FB): Utah 1 (FB ≤ 5%), Utah 2 (5% < FB ≤ 20%), Utah 3 (20% < FB ≤ 35%) or Utah 4 (FB > 35%). As summarized in Table 1, the patients were labelled 1 to 6 in decreasing order of their FB.

### Fibrosis implementation in the computational LA models

To study how the RDs localize in fibrotic regions and design a novel tool to identify regions where they stabilize relative to the patient-specific fibrosis distribution, we modelled patchy fibrosis as regions of slow conduction [16]. Furthermore, in the fibrotic tissue the decrease in conduction velocity (CV) was set in proportion to the recorded LGE MRI intensity, as reported in *in-vivo* experimental studies [32]. This was achieved by altering the diffusion coefficient, *D*, in these regions proportionally to the IIR. Thus, healthy tissue with an IIR < 1.08 had *D* = [32]1 mm^2^/ms, dense fibrotic tissue with IIR ≥ 1.24 had *D* = 0.017 mm^2^/ms (which is ~83% lower than the value *D* value of healthy tissue) and in the fibrotic BZ with 1.08 < IIR < 1.24 *D* had intermediate values calculated via linear interpolation between 0.1 and 0.017 mm^2^/ms (**Fig 2**). Note that the values of *D* chosen for modelling the dense fibrotic region and the surrounding border-zone were not validated due to a lack of experimental data. However, a correlation between decrease in conduction velocity (which is proportional to *D*) with increase in IIR has been reported [32]. Therefore, our approach of gradually decreasing CV with increasing IIR across fibrosis regions is in agreement with patient studies.

The value of *D* for dense fibrotic regions was not set to 0, since there is no experimental evidence suggesting that dense fibrotic regions are completely non-conductive. In control models without fibrosis, the patient-specific LA geometry was preserved, but *D* was set to 0.1 mm^2^/ms for all patients. In the resulting LA models, CV in healthy tissue was 0.6 m/s, while in fibrotic tissues CV ranged between 0.1–0.6 m/s.

### AF simulation protocol and data analysis

Each patient-specific LA model was paced 7 times at a basic cycle length (BCL) of 130 ms at different locations near the PVs. A plane wave was initiated in 20 ms after the last ectopic beat. The interaction between the plane wave and the ectopic beats allowed for the generation of RDs. By varying the pacing site, the direction of the plane wave and the time interval between them, RDs were initiated in 8 to 12 different locations in each LA model. In each simulation, we (i) tracked the RD tips for a duration of 6 s, (ii) identified regions where they were located in the last 1 s of the simulation, and (iii) constructed a RD tip frequency map by recording the number of times each tissue voxel was visited by the RD tips over the course of the simulation.

For each patient, all the RD tip frequency maps obtained in the AF simulations were combined to construct patient-specific RD probability maps, showing the relative frequency with which each voxel was visited by the RDs (see S1 Fig). The normalised tip probability maps were thresholded and locations with a normalised probability over 0.2 were identified as TAs. The threshold value of 0.2 was computed using a standardised approach of taking 2-standard deviation from the mean of the normalised tip probability map. In future, this value will be validated using EAM data.

The simulations and the analysis process were performed in all the 6 LA models with and without the presence of fibrotic tissue. The TAs identified from both the cases were compared using the Dice score [33], a standard metric for measuring the degree of spatial overlap.

### Catheter ablation strategies

**Fig 4:**
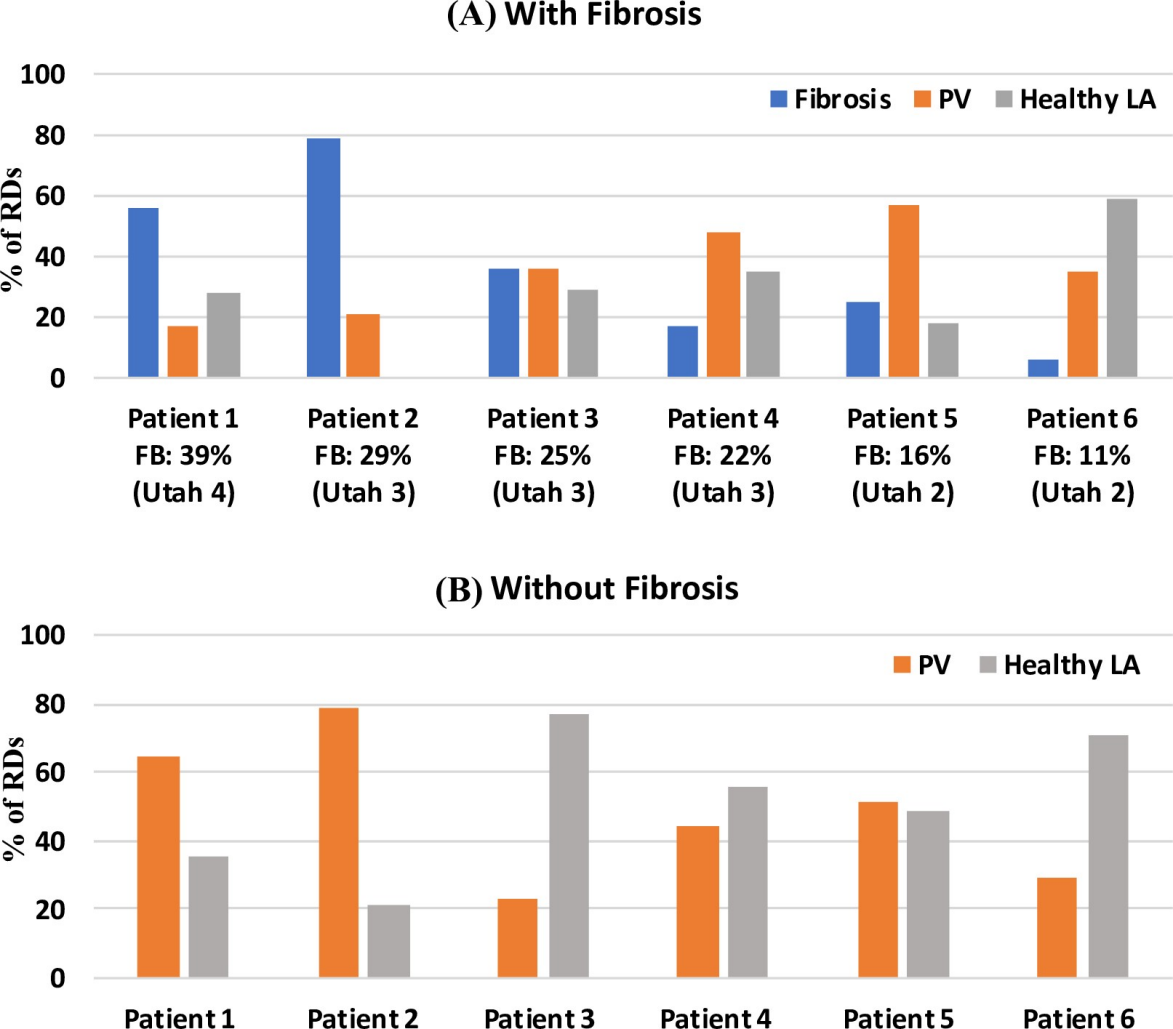
Regions of RD stabilisation in patient-specific LA models. The image shows bar chart with percentage of RDs found in different LA regions (blue: fibrosis, orange: PVs and grey: healthy LA tissue) after 6s of the simulation in the 6 patient-specific LA models with (A) and without fibrosis (B). A) In Utah 2 patients, the primary location for the RDs is the PVs. In Utah 3 patients, RDs are distributed between fibrotic regions and the PVs. In Utah 4 patients with severe fibrosis, the primary location of RDs are the fibrotic regions. FB: fibrotic burden.

**Fig 5:**
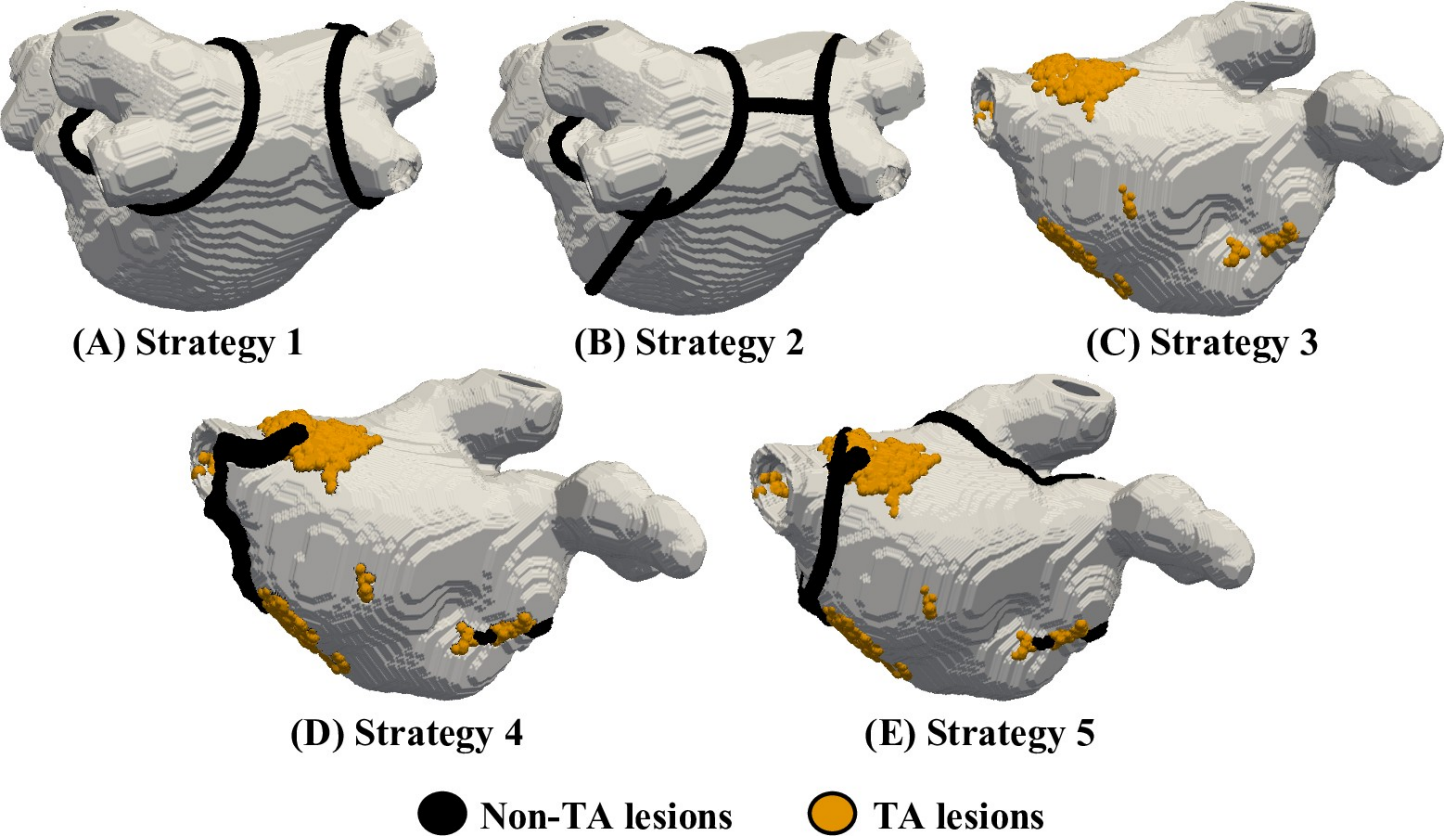
CA strategies tested using virtual ablation platform. (A) Strategy 1: Circumferential PVI, (B) Strategy 2: PVI plus additional linear lesions at the LA roof and a line joining the PV to the MV, (C) Strategy 3: ablation on the TAs, (D) Strategy 4: TA guided ablation with additional linear lesions leading to unexcitable boundaries (PV and MV) and (E) Strategy 5: TA guided ablation with linear lesions (Strategy 4) plus PVI (Strategy 1).

As a proof-of-concept, virtual CA was simulated in a subset of the LA models: Patient 2 and 3 with a FB in Utah 3 category. The choice of these patient models was motivated by the highest correlation found between the RD location and fibrotic region in **Fig 4**A. However, Patient 1 was not ablated as the atrial tissue was severely fibrotic (Utah 4), and ablation would not leave much healthy tissue. The CA lesions were modelled as transmural regions of unexcitable tissue with a cylindrical shape with a diameter of 3 mm [34] to account for the catheter tip shape. The continuous CA lesions were implemented using five strategies; Strategy 1 and 2 are used clinically [5], Strategy 3 is based on our model predictions and Strategies 4 and 5 are combinations of the model predictions with clinical strategies. Further details of each strategy are provided below:

1. Strategy 1: PV isolation (**Fig 5**A)
  The 2 right and 2 left PVs were electrically isolated by a continuous set of ablation lesions encircling the PVs and isolating them from the remaining LA body.
2. Strategy 2: PV isolation with linear lesions (**Fig 5**B)
  If the RDs still persisted after the application of Strategy 1, additional linear lesions were applied: one on the LA roof to connect the left and right circular PVI lesions and another to connect the roof lines with the mitral valve (MV) opening.
3. Strategy 3: TA guided ablation (**Fig 5**C)
  Ablation lesions were applied to the voxels within the TAs identified in the patient-specific AF simulations. This approach has similarities to DECAAF II, an ongoing prospective multicentre randomised trial in which all fibrotic regions identified from LGE-MRI are targeted by CA. However, our approach only targets TAs that typically are located near fibrotic regions but are much smaller than the entire area of fibrosis.
4. Strategy 4: TA guided ablation with linear lesions (**Fig 5**D)
  If the RDs still persisted after the application of Strategy 3, additional linear lesions were applied to join the TAs to the nearest anatomical boundary–the PVs or the MV.
5. Strategy 5: TA guided ablation with linear lesions and PVI (**Fig 5**E)
  If the RDs still persisted after the application of Strategy 4, additional Strategy 1 (PVI) was applied.

All CA lesions were applied after 6s of the AF simulation, and the ability of these lesions to terminate the existing RDs within the following 2 s was analysed. The CA strategy was considered successful if either AF converted to atrial tachycardia (AT) or all RDs were completely terminated. Here we referred to AT as a single RD anchored around scar tissue or an anatomical boundary such as the MV or the PVs. The mean frequency (MF) of activations in the LA model before and after CA was recorded for all the strategies and conversion of AF to AT was evaluated by reduction in the MF [35]. The MF was computed by averaging, across all voxels in the LA geometry, the dominant frequency calculated during or the last 1s of the simulation. Moreover, for TA guided CA strategies 3 and 4, we also tested if the applied CA lesions prevented AF inducibility. To achieve this, the protocol used for AF initiation was repeated again after the application of the CA lesions.

## Result

### Patient-specific fibrosis distribution in 3D atrial models

The LGE-MRI intensity-based reconstruction yielded different fibrosis distributions in all 6 AF patients, as shown in **Fig 3**. Large variations in the extent and severity of fibrosis were observed across the LA models (Table 2). Amongst the PsAF patients, P1 had the highest FB of 39% and was classified into Utah 4 category, and P2 with 29% FB was in Utah 3 category. In the PAF patients, P3 and P4 with a moderate FB of 25% and 22%, respectively, were in Utah 3 category; P5 and P6 with mild FB were in Utah 2 category.

**Table 2:** Characteristics of the 6 AF patients whose LA models were used in the study.

Patient	% FB	% Volume of largest patch	% FB of PVs	% FB of LA wall	% Volume* of dense tissue
P1	39	37.95	28.84	71.16	12.98
P2	29	18.27	42.86	57.14	6.38
P3	25	16.63	35.31	64.69	6.37
P4	22	8.54	63.28	36.82	8.18
P5	16	6.42	42.10	52.10	6.48
P6	11	4.08	64.53	35.47	0.24

Properties of patient-specific fibrosis distributions. The size of the primary fibrotic patch decreases with the fibrotic burden (FB).

* All volumes are reported as percentage of the atrial wall volume to standardise the measurements.

In addition to differences in severity, a large variation in the size distribution of the fibrotic patches was also observed across all patient-specific LA models, as summarised in Table 2. These estimates suggested the size of the largest fibrotic patch recorded per patient increased with increasing the overall FB. Moreover, each individual patient LA was characterised by the presence of a primary fibrotic patch with a volume significantly greater than the secondary surrounding patches. The distribution of fibrotic regions between the PV regions and remaining LA wall was recorded across the patient-specific LA models. Here the PV region was defined as a 3 mm spherical region around each PV opening. In P1 (Utah 4), P2 and P3 (both Utah 3) with the highest FB, the majority of the fibrotic regions were at the LA wall. While, as the FB decreased the fibrotic regions were mostly located in the PV region.

### Atrial fibrosis influences the distribution of RDs in patient-specific LA models

In the patient-specific LA models, AF was successfully initiated in all 6 patients using the cross-field protocol with and without the presence of fibrosis. The RD initiation protocol was applied to each patient-specific LA model, either with or without fibrosis, and resulted in the formation of either 1–2 RDs in the first 1s of simulation. In the following 5s, these RDs meandered and eventually stabilised at distinctive locations in the atrial wall. In models without fibrosis, these locations were influenced by the atrial geometry only, while in the presence of fibrosis, these were dependent on both, the patient-specific fibrosis distribution and geometry. To get a better understanding of how the distribution of RDs across the patient-specific geometries was affected by the overall FB, we classified the RDs in every model into 3 groups according to the region where they stabilised in the last 1s of the 6s-long simulations. The outcomes of this classification are summarised in **Fig 4** with a bar plot showing the % of the total RDs anchored to: (i) fibrotic patch in blue, (ii) PVs in orange and (iii) healthy non-PV tissue in grey for all 6 patient-specific LA models with (A) and without fibrosis (B).

#### Persistent AF Patients (Utah 4 &3: FB > 25%)

In these patients (**Fig 4**A, Patient 1 and 2), the RDs stabilised primarily at fibrotic regions (P1: 56%, P2: 79%), compared to lower probabilities of stabilisation in the PVs region (P1: 17%, P2: 21%). However, when the same LA simulations were repeated for these patients without fibrosis, the RDs stabilised primarily at the PVs (**Fig 4**B, P1: 65%, P2: 79%). This suggests that in these PsAF patients with very high FB, the presence of large quantities of slow conducting fibrotic tissue influenced the RD locations, facilitating their anchoring to the fibrotic patches instead of the PVs.

#### Paroxysmal AF Patients (Utah 3: 20% < FB < 25%)

In this category of AF patients with intermediate levels of fibrosis (Patients 3 and 4), the RDs stabilised with similar probability in all three regions (**Fig 4**A). Comparing the distribution of RDs across different regions in simulation with (**Fig 4**A) and without fibrosis (**Fig 4**B) suggests that fibrosis around the PVs in these patients facilitated anchoring of RDs to the PVs. Moreover, despite having similar FB, the variation is distribution of the RDs across the patient-specific LA models (**Fig 4**A, P3 & P4) can be explained by the difference in spatial distribution and sizes of fibrotic patches in the respective LA models. In Patient 4, the majority of RDs anchored to the PVs (**Fig 4**A, P3: 38%, P4: 48%), as the amount of fibrosis in regions adjacent to the PVs were relatively greater than Patient 3 (Table 2, P3: 35% and P4: 63%).

#### Paroxysmal AF Patients (Utah 2: FB < 20%)

In this category of AF patients with the lowest FB (**Fig 4**A, Patients 5 and 6), the number of RDs anchoring to PVs (P5: 57%, P6: 35%) was higher than that in the fibrotic regions (P5: 25%, P6: 6%). This agrees with clinical observations that in PAF patients with lower FB, RDs are likely to be located at the PVs. In the absence of strong fibrosis effects, the distributions of RDs across different region in simulation with (**Fig 4**A) and without fibrosis (**Fig 4**B) showed similar trends. In Patient 5, the PV region had the highest percentage of RDs either with or without fibrosis, and in Patient 6 the LA wall had the highest percentage of RDs either with or without fibrosis. The results suggest that other factors, such as the LA and PV shape, specifically changes in curvature of the underlying geometry, can influence the RD dynamics [36,37].

### Patient-specific fibrosis determines the RD anchoring locations

In the previous section, we reported the overall distribution of the RDs across different regions of the atrial wall in the patient-specific LA models and found that RDs are most likely to be present at fibrotic regions in patients with FB from Utah category 3 and 4. However, the basic knowledge that RDs are present at fibrotic regions is insufficient to guide CA–we need to pinpoint the exact locations within the fibrotic patches where the RDs are localised. This warrants a detailed analysis of the mechanisms that affect RDs anchoring to specific fibrotic patches.

**Fig 6:**
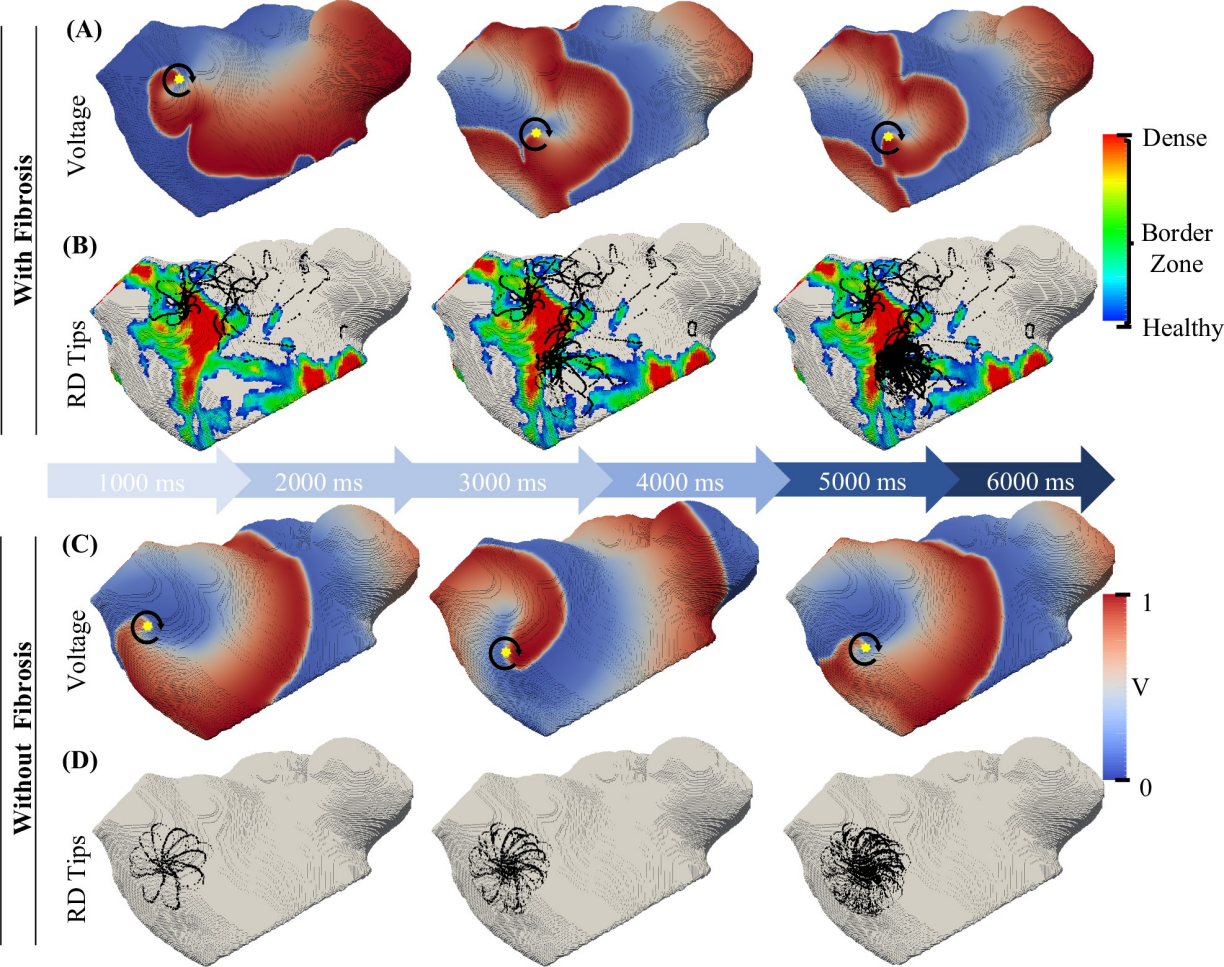
The effect of fibrosis on the RD dynamics in a patient-specific LA model. A & C: the colour-coded voltage maps (normalised, so that 0 is rest state and 1 is maximal activation) in the LA of Patient 3 over 6s of simulation with the yellow dot indicating the RD tip and the arrow pointing to the direction of rotation. B & D: the corresponding RD trajectories (in black) superimposed on the fibrosis distribution. In simulations with fibrosis (A-B), the primary RD drifts around and across the fibrotic patch within the first 1s and thereafter stabilises at a distinctive location within the fibrotic BZ. However, in the same simulation without fibrosis (C-D), the primary RD remains at the location of its initiation.

To illustrate the concept, **Fig 6**A, B shows voltage maps and RD tip locations in a simulation of AF maintained by a single RD anchored to a fibrotic patch in the anterior LA wall. After the initial meandering, the RD tip (represented by a yellow dot) remained at a specific location inside the fibrotic patch for the entire duration of the simulation. Similar RD behaviour has observed in other patients. However, the specific location where the RDs anchored to fibrotic regions was dependent on the size and distribution of fibrotic patches. Moreover, the same protocol when repeated in the absence of fibrosis resulted in the formations of RDs that either stabilised at the region where they were initiated (as in **Fig 6**C and **6**D) or they drifted towards the PVs and MV, under the influence of the underlying curvature of the atrial geometry.

**Fig 7:**
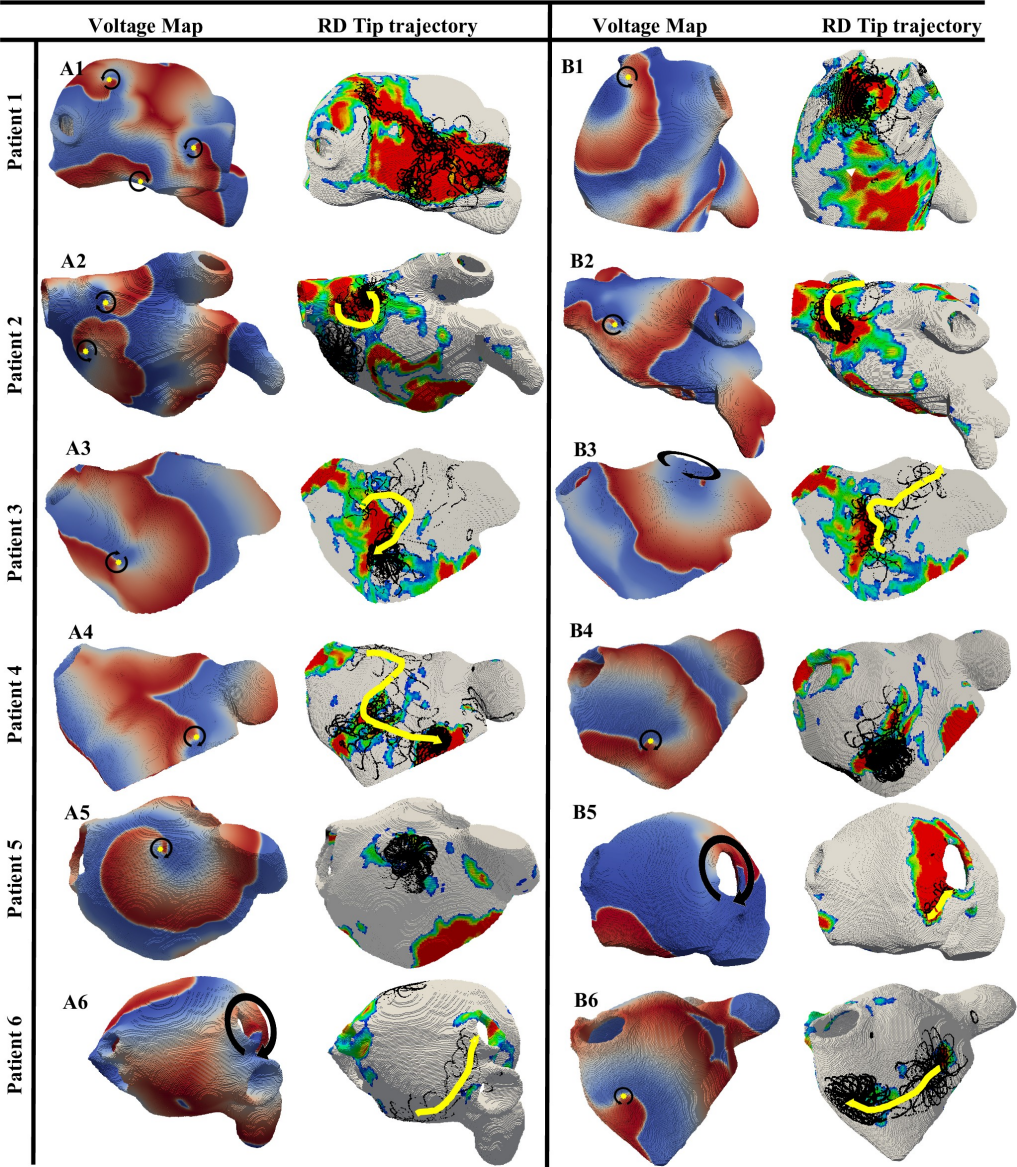
The anchoring of RDs to fibrotic patches in patient-specific LA models. For all the 6 AF patients (rows), sections A and B show the AF simulation outcomes for 2 different RD initiation sites. In each section, on the left are the colour-coded voltage maps and on the right, the respective RD trajectories (black) superimposed on the fibrosis distributions. The yellow arrows indicate the direction in which the RD drifted over 6s of the simulation. The colour map used in the figure is the same as **Fig 6**.

Further examples of RD trajectories under the influence of fibrotic patches for each of 6 patients are presented in **Fig 7**. Here, in Patient 1 from Utah 4 category, more complex activation patterns in the voltage map were observed in addition to anchoring of RDs to fibrotic patches (**Fig 7**, A1). This was due to the breakdown of the initial single RD into three new ones, which all stabilised at various locations around the largest fibrotic patch and surrounding BZ. In Patients 2, 3 and 4 from Utah 3, the RDs were found to stabilise at distinct regions of fibrotic patches (**Fig 7**, A2-A4 and B2-B4). In Patients 5 and 6 from Utah 2, the fibrotic patches were much smaller compared to those in patients from Utah 3 and 4 categories. Here, the RDs often stabilised between two small fibrotic patches (**Fig 7**, A5 and B6), and if fibrotic patches were in regions surrounding the PVs (**Fig 7**, A6 and B5), fibrotic tissue aided the RD moving towards and anchoring to the PV opening. All these examples highlight the patient-specific characteristics of fibrotic patches play an important role in detaining the dynamics and ultimate anchoring locations of RDs.

### Targets for ablation identified from patient-specific LA models

In the patient-specific LA models, as presented in the previous section, the RD anchoring locations were strongly dependent on the highly heterogeneous patterns of fibrosis distributions across the patient-specific LA models. Moreover, the anchoring locations were specific to individual patches. Therefore, by analysing all the RD tip trajectories obtained from numerous locations in each of the 6 patient-specific LA models (using the protocol illustrated in S1 Fig), we identified specific locations near fibrotic regions where the RDs were most likely to be found–the areas of high probability of the tip localisation–the TAs.

**Fig 8:**
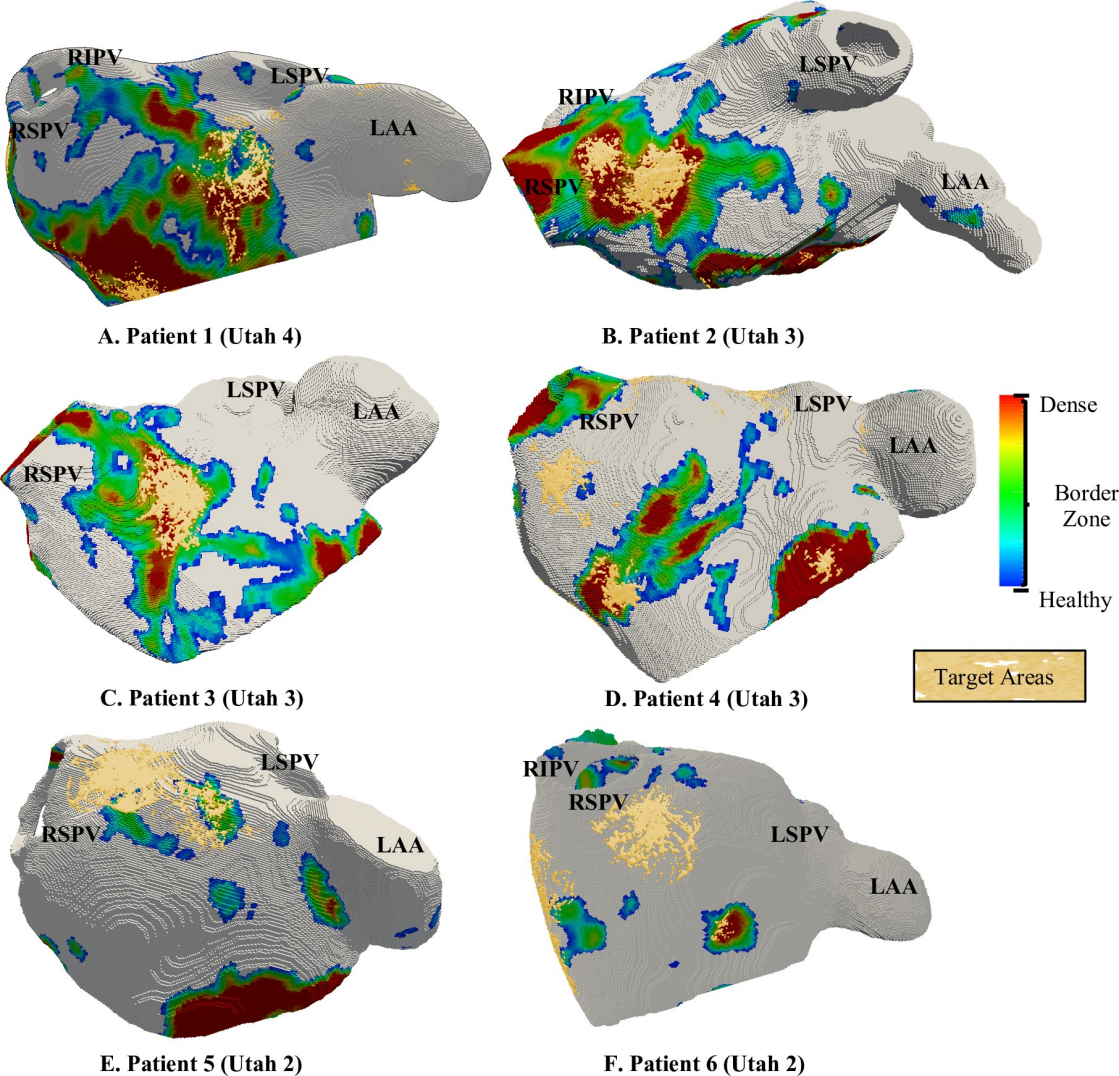
Patient-specific RD location maps–potential CA targets. The images show colour-coded fibrosis distributions in the 6 patient-specific LA models (A-F), with the TAs superimposed. In Utah 3 and 4 categories (Patients 1, 2 and 3), TAs are seen at specific locations within fibrotic patches and at their BZs. In Utah 2 category (Patients 5 and 6), TAs are seen on the LA wall near small patches. Additionally, the TAs identified from simulations performed without fibrosis in the same LA model are presented in S3 Fig.

**Fig 9:**
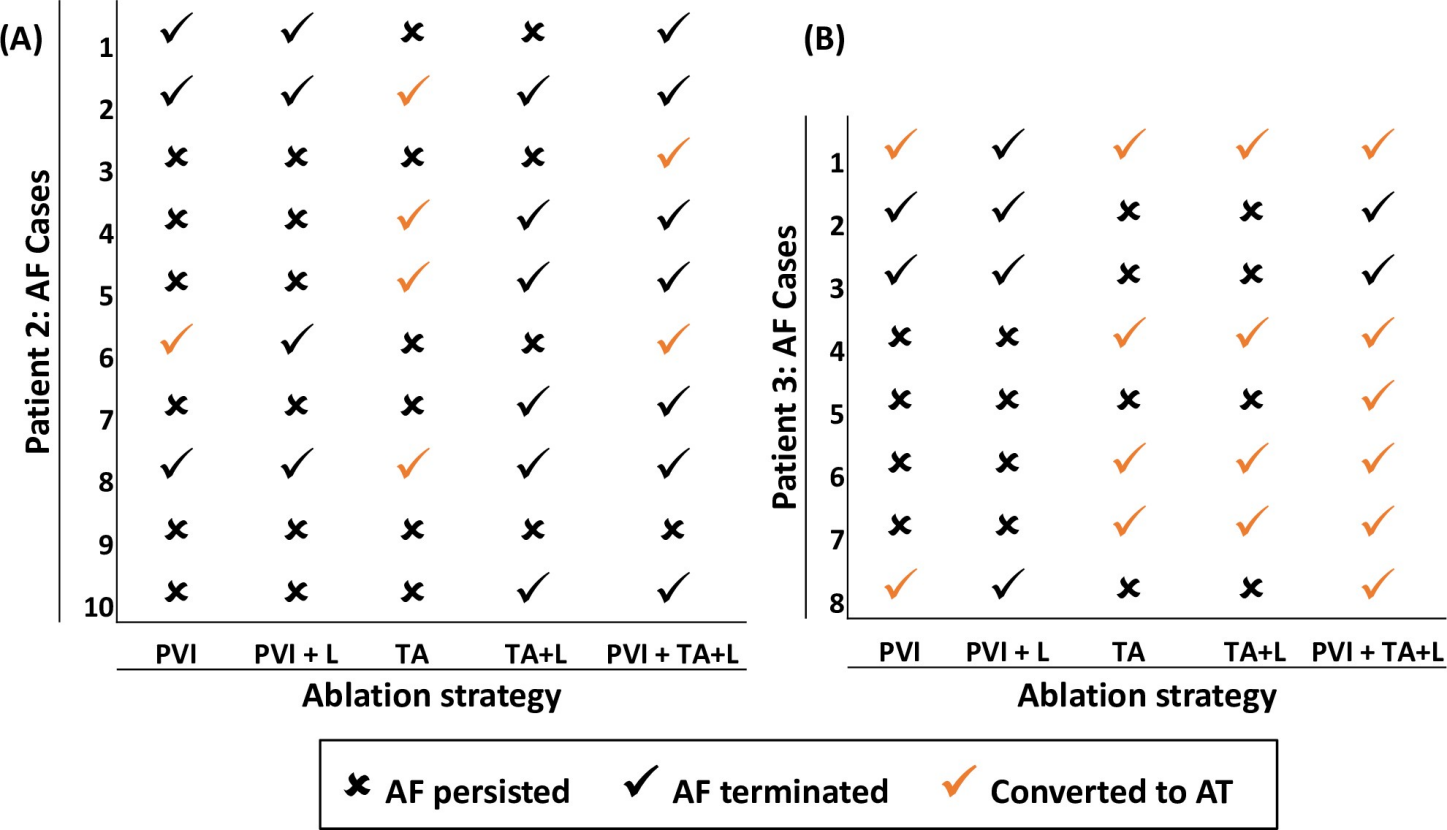
Comparison of the fibrosis burden to TA volume of in each patient-specific LA models. A: distribution of TAs across different regions of the atrial wall; dense fibrotic region (black), border zone (BZ—dark grey) and healthy tissue (light grey). The majority of TAs lie in the fibrotic region in Patients 1, 2 and 3 (FB > 25%, Utah 4 and 3), compared to much lower numbers in Patients from 4, 5 and 6 (FB < 25%, Utah 3 and 4). B: bar chart with FB (blue) and volume of all identified TAs (orange) in each model. The difference between FB and TA volume decreases with the decrease in Utah score, showing that the predicted TAs can be most efficient in improving CA in Utah 3 and 4 patients.

The TAs obtained in all 6 patient-specific models are shown in **Fig 8**. An example of TA distribution computed from the normalized RD tip probability maps for the LA model of Patient 2 is provided in the S2 Fig with different values of threshold. The TAs are computed by thresholding the probability maps such that only locations that are most likely to be visited by the RDs are captured. On average 5.33 ± 2.94 TAs were identified per patient-specific LA model. Moreover, by further analysing the distribution of the TAs (**Fig 9**A), we found that a higher percentage of TA were located within the fibrotic tissue region in patients in Utah 3 and 4 categories compared to patients in Utah 2 category. However, all TAs were relatively small and the total volume of TAs was much lower than that of fibrosis in all patients (**Fig 9**B). The difference in the volume of fibrotic tissue and TAs was highest in Utah 4 patients, and lowest in Utah 2 patients, where RDs often stabilised around the PVs rather than fibrosis.

Additionally, we repeated all patient-specific LA simulations without fibrosis (S3 Fig) and compared the TA locations to those in the respective LA models with fibrosis using the Dice score, a standard metric for the degree of spatial overlap. We did not find any link between the number of RDs and the presence of fibrosis. This could be due to the use of cross-field protocol for the RD initiation, which makes the number of RDs independent of the presence of fibrosis. However, the Dice score comparing the TAs between these two models was found to decrease with increasing FB. Thus, Patient 6 (FB: 11%) had Dice score of 0.52 compared to Patient 1 (FB: 39%), who had an extremely low Dice score of 0.06. This means that, in the presence of large fibrotic areas, the RDs rarely were found in the same locations where they would be found in the absence of fibrosis. These results provide further evidence for the role of fibrosis in determining the RD locations. However, we did not find any link between the number of RDs and the presence of fibrosis. This could be due to the use of cross-field protocol for RD initiation in both the models which makes the number of RDs independent of the presence of fibrosis.

### Ablation strategies

**Fig 10:**
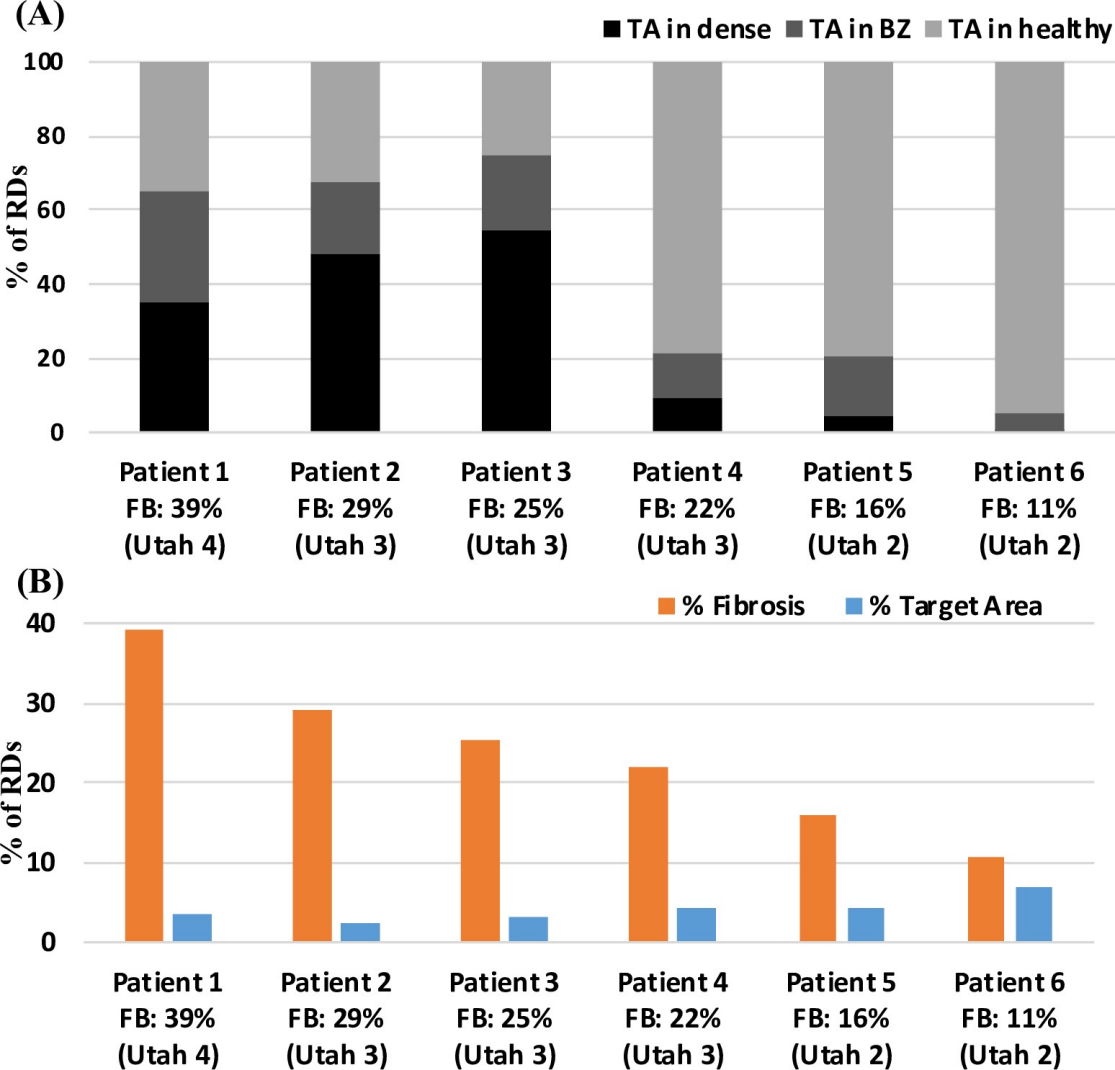
The outcome of CA using different strategies in Patient 2 (A) and Patient 3 (B). The summary of MF recorded for these cases are presented in the S1 Table.

**Fig 11:**
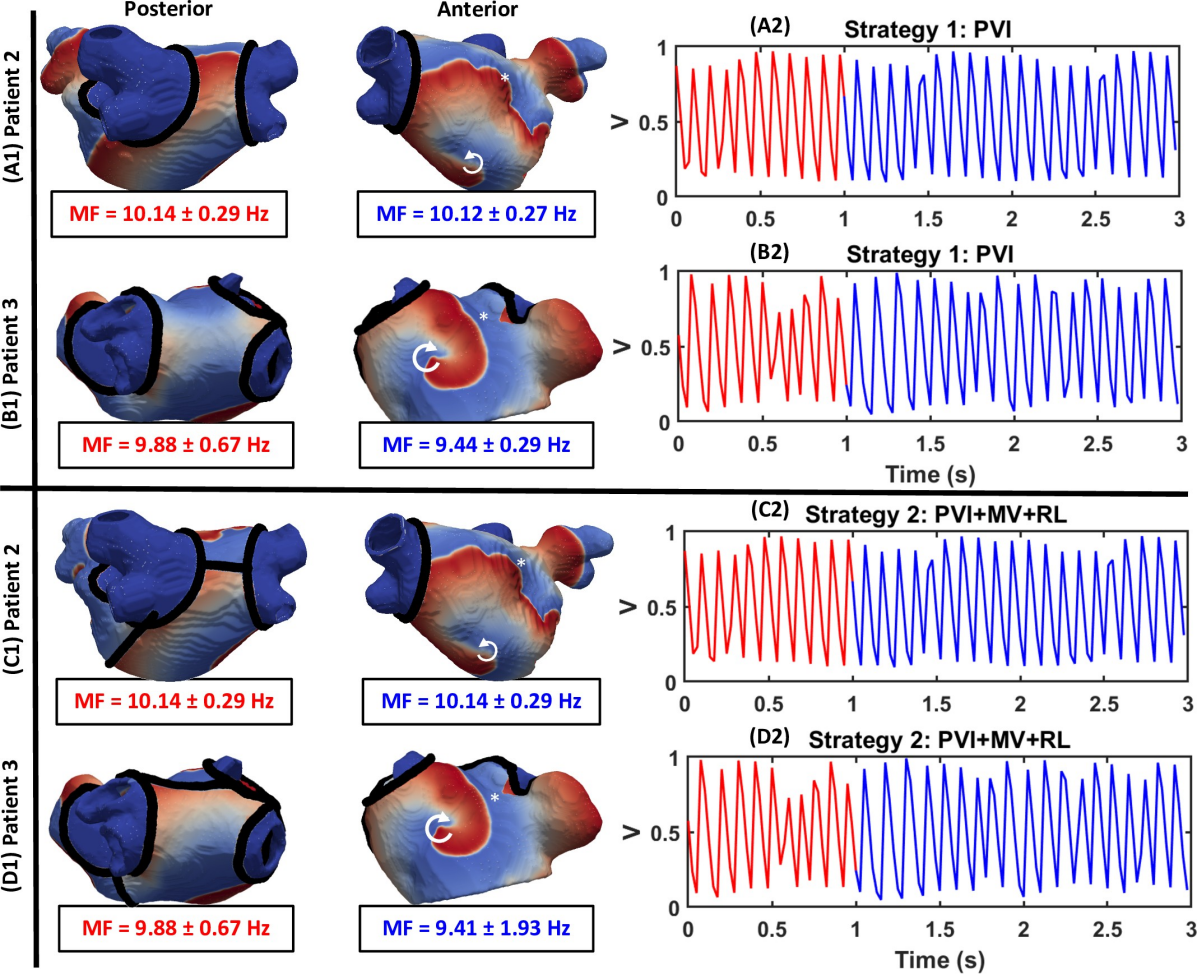
CA with the clinical used ablation Strategies 1 and 2 failed to terminate AF in the majority of cases. Left: Voltage maps showing the behaviour of RDs after: (A1-B1) Strategy 2 and (C1-D1) Strategy 2 in Patient 2 and 3, respectively. Right: Transmembrane voltage at the point indicated by * in the respective LA models, plotted before (red) and after (blue) CA. Both strategies failed to terminate RDs in all cases and MF remained unaffected. Here and in the next figure, the white arrow indicates the directions of RD movement.

**Fig 12:**
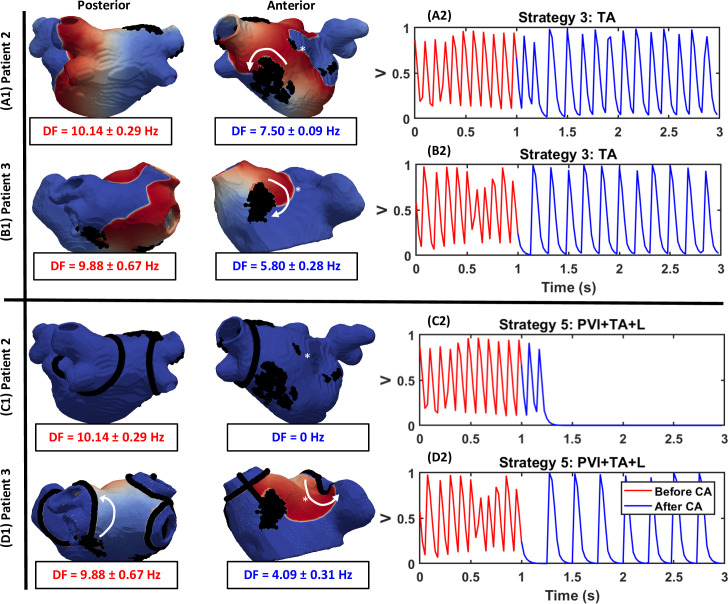
Target area guided ablation with Strategy 5 successfully terminated AF. Voltage maps showing the behaviour of RDs after CA of: (A1-B1) TAs and (C1-D1) TAs plus linear lesions joining them to the PVI lesions and the mitral valve (MV) in Patient 2 and 3, respectively. Panels on the right (A2-D2) show the voltage at the point indicated by * in the respective LA models, before (red) and after (blue) CA. Out of the two strategies, PVI plus TA guided ablation successfully terminated AF in both patients.

The outcomes for all virtual CA strategies in both patients are summarised in **Fig 10** and described in detail below in regard to **Fig 11** and **Fig 12**.

Ablation of the PVs (in Strategy 1) failed to terminate RDs in the majority of cases (P2: 6/10, P3: 4/8). As shown in **Fig 11** (A1 and B1), RDs located far from the PVs were unaffected. These RDs persisted even after additional linear lesions were applied at the roof and the MV (**Fig 11**, C1 and D1, in Strategy 2). In the few cases where AF did terminate, the RDs were present inside or near the isolated PV regions. After CA, these RDs either anchored to the lesions encircling the PVs (P2: 1/10, P3: 2/8) resulting in AT or terminated (P2: 3/10, P3: 2/8).

Ablation of the TAs was performed using Strategies 3, 4 and 5. In Strategy 3, the application of CA lesions on top of the TAs either resulted in the RDs anchoring around the newly formed ablation scar (**Fig 12**, A1 and B1) and AF converting to AT (P2: 4/10, P3: 4/8) or had no effect (P2: 5/10, P3: 4/8). Examples of the conversion from AF to AT in Patient 2 and 3 are shown in **Fig 12**, A2 and B2. Here, MF dropped by 26% and 41%, respectively. However, Strategy 4 connecting the TAs with linear ablation lesions to the nearest boundary (the PVs or MV) terminated AF in the majority of the cases (P2: 6/10, P3:4/8). In the case of Patient 2, all RDs were terminated (**Fig 12**, C1), while in Patient 3, the RDs anchored to scar after ablation tissue converting AF to AT (**Fig 12**, D1), with a reduction in MF by 59%. In Strategy 5, application of PVI in addition to Strategy 4 resulted in a further increase of AF termination rate in both patients (P2: 9/10, P3: 8/8). However, it should be noted that the percentage of ablated tissue was increased with additional lesions. For example, the percentage of ablated tissue in Patient 2 was 6%, 7% and 14% for strategies: 1, 3 and 5, respectively.

AF recurrence after CA is a huge clinical problem. In order to check for AF recurrence, we tested for AF inducibility after virtual CA Strategies 3 and 4. To achieve this, the protocol used for AF initiation (described in the Method section) was repeated after the application of the ablation lesions from 10 different sites. CA performed using Strategy 3 did not prevent the RDs from being initiated in either patient. In Patient 3, for all 10 cases of initiation, the RDs anchored to scar tissue resulting in AT. In Patient 2, 8 out of 10 cases resulted in AT and in the other 2 cases AF was induced. Finally, after using Strategy 4, neither AF nor AT was inducible in the LA model of Patient 2 in 9 out of 10 cases, while in Patient 3, all 10 cases resulted in AT with a lower MF (~ 5 Hz) compared to Strategy 3.

## Discussion

This study developed a novel image-based computational workflow for the identification of patient-specific locations of RDs sustaining AF. Specifically, we: 1) developed 3D LA models with patient-specific geometry and distribution of fibrosis obtained from LGE-MRI of 6 AF patients, 2) applied the models to explore the dynamics of RD stabilisation in the presence of slow-conducting fibrotic patches, 3) identified patient-specific TAs for CA using the RD locations, relative to the distribution of fibrosis and 4) evaluated AF termination by simulating several ablation protocols, including TA-guided ones.

The main observations of this study were as follows: (i) in AF patients from Utah 4 and 3 with high FB (>25%) RDs were more likely to be found at fibrotic regions compared to PVs (**Fig 4**A), (ii) RDs anchored to specific regions within the atrial walls–TAs identified from RD tip probability maps (**Fig 8**), (iii) a higher percentage of TAs were located within the fibrotic tissue region in patients in Utah 3 and 4 categories compared to patients in Utah 2 category (**Fig 9**A), and (iv) performing virtual CA of the TAs and connecting them with linear lesions to the nearest PVs or MV had superior anti-fibrillatory effect compared to ablating the TAs alone, as well as compared to clinically accepted strategies such as PVI. Fibrosis-based ablation in Utah 3 and 4 patients would result in extensive damage of the atria, and therefore tailoring CA strategies to TA regions predicted by the image-based models may help improve CA therapy.

These results agree with recent computational studies that provided the first evidence for the anchoring of RDs near atrial fibrotic regions [15,16,38]. McDowell et al. [14] first showed that in patient-specific LA models with fibrosis the localization of RD tips during AF (linked with the RD movement) was independent of the pacing locations from which AF was induced. Zahid et al. [15] then used similar models to demonstrate that AF was perpetuated by RDs that persist in spatiallsy confined regions, with the latter constituting boundary zones between fibrotic and non-fibrotic tissue. Finally, Morgan et al. [16] explained the mechanisms of RD anchoring to the fibrotic BZ by slow-conducting properties of the latter, which enabled the development of re-entrant circuits within relatively small regions. The current study builds on these results, exploring how the specific size of fibrotic patches and their BZ provides a more favourable substrate for the RDs, and therefore provides the basis for the creation of patient-specific maps of the RD locations.

### Influence of atrial fibrosis on the distribution of RDs

Our computational results are also in good agreement with clinical studies that have reported high levels of arrhythmogenic activity around patches of atrial fibrosis [11] and targeted low-voltage areas, identified from atrial mapping and associated with the presence of fibrosis, to improve CA outcomes [12,13]. Moreover, a recent clinical study has directly correlated the patient-specific enhanced LGE MRI areas with locations of RDs recoded using electrocardiography [18]. Our image-based LA models provide in-depth insights into the links between fibrosis properties and RD behaviour, which are virtually impossible to achieve even using advanced imaging systems in a clinical setting.

We also found a strong link between fibrosis burden and the probability of RDs anchoring to fibrotic regions. Thus, in Patient 1 with 39% fibrosis burden (Utah 4 category), the fibrotic regions were identified as the primary clustering location of RDs compared to PVs. In Patient 6 with low fibrosis burden of 11% (Utah 2 category), RDs were mostly anchored around the PVs. These results are in agreement with clinical studies which have correlated the success of PVI to fibrosis burden [10] and computational studies that showed the sustenance of AF primarily in patient from Utah 4 category [14]. Hence, the higher FB translates into a higher probability of RD localization in fibrotic regions and may increase benefits of fibrosis-based CA compared to PVI in these patients.

However, it is worth noting that RDs locations between patients with similar fibrotic burden can be critically influenced by a specific fibrosis distribution. Thus, for Patients 3 and 4 (both from Utah 3 category) with a similar fibrosis burden, there was significant difference in the locations of RD stabilization (**Fig 4**). This could be explained by the differences in the spatial distribution of fibrosis among the two patients, leading to RDs stabilization in only a few patient-specific regions (**Fig 8**). Similar effects of fibrosis on the RD dynamics have been reported in studies by Zahid et al. [15] and Morgan et al. [16]. These results provide mounting evidence that unique patient-specific distributions of fibrotic tissue can determine RDs locations and help identify TAs for ablation.

Other studies have also incorporated the electrotonic effects of fibroblast-myocyte coupling [16], ionic changes due to paracrine effects [15] and represented the deposition of collagenous fibres using the percolation method [39] or discontinuous finite elements [40], and showed collocation between RDs and fibrotic boundary zones [16,41]. Computational studies that compared different fibrosis modelling methodologies have reported that these additional factors further facilitated the anchoring of RDs to the fibrotic areas and their BZ [42,43]. This may explain why those simulations found a larger number of RDs located at the fibrotic BZ compared to ours. However, slow conduction in fibrotic BZ has been shown to be more important for RD anchoring mechanisms than myocyte-fibroblast coupling or ionic remodelling [16]. We believe that, although our representation of fibrotic tissue is simple, it is adequate for patient-specific models derived from clinical imaging data in the absence of more-detail patient recordings.

Note also that Vandersikel et al. [44] have demonstrated a new mechanism that facilitated the anchoring of RDs to fibrotic regions in ventricular tissue via dynamical reorganization of the excitation pattern. However, we were unable to identify such mechanism in our atrial models. Potential reasons include electrophysiological differences between the chambers, as well as differences arising from the variable methodologies adopted for the modelling of fibrotic regions. In our study, patchy fibrotic regions had low conductivity inversely proportional to the underlying LGE intensity, while Vandersickel et al. [44] modelled fibrotic regions with electrically uncoupled unexcitable nodes depending on the LGE intensity. Adoption of uniform methodologies and comparison of RD anchoring mechanisms in the atria and ventricles can shed further light on the general fibrillatory mechanisms in the heart.

### Virtual ablation on the predicted TAs

Virtual CA of the predicted TAs was performed on a subset of patient-specific LA models and its success was compared with existing clinical CA strategies.

We tested two clinical strategies: (1) PVI and (2) PVI with additional linear lesions at the LA roof and MV. The former is considered a cornerstone of CA in PAF patients [45], while the latter has been used in combination with PVI in patients with chronic forms of AF [46]. We then compared the outcomes of these two CA strategies with three TA guided ablation strategies: (3) ablation of the TAs only, (4) ablation of the TAs with additional application of linear lesions joining the TAs to the nearest boundary (the PVs or MV), and (5) the ultimate strategy that combined TA guided ablation with PVI and a connecting set of linear lesions.

Our simulation results demonstrated that in cases where RDs are present in regions far away from the PVs, clinical CA strategies (Strategies 1–2) were unable to terminate AF. Virtual ablation of TAs alone (Strategy 3) resulted in the stabilisation of meandering RDs in the vicinity of ablated regions, and the conversion of AF to AT with a lower MF (**Fig 12**A and **12**B). Similar findings have been reported in a study by Bayer et al. [47], where CA directly targeted the RD tip. Although Strategy 3 reduced MF to about 6 Hz in some simulations, in most cases the patients remained in rapid AT with MF of up to 8 Hz (see S1 Table). Strategy 4 successfully terminated AF in majority of these cases for both patient-specific LA models either by eliminating the RDs and AF or by converting AF to AT (P2: 60%, P3: 50%). Strategy 5, combining Strategies 1 with Strategy 4, resulted in an increase in AF termination rate (P2: 90%, P3: 100%) compared to Strategies 1 only (P2: 40%, P3: 50%).

Note that a recent clinical study by Calvo et al. [48] have used a similar approach, where ablation on RD domains identified using electroanatomic mapping (EAM) was performed with limited linear lesions joining the identified RD domains with the unexcitable boundaries (PVI lesion in the LA). Their results showed a reduction in dominant frequency and acute termination to sinus rhythm in 15% of persistent AF patients, and at 1 year follow up showed 70% of patients were free from AF. Our simulation results are consistent with these findings and can explain the mechanism underlying its success. Note also that Calvo et al. relied on EAM to identify the RD domains, which can be unreliable due to limitations of EAM technology (e.g., poor atrial coverage and mapping resolution). Our personalised image-based computational models can enable the identification of these regions with greater accuracy, which further highlights the potential of such models for improving the efficiency of CA in chronic AF patients.

The image-based computational workflow presented in this paper is a promising tool which can build up on mechanistic knowledge and help improve CA therapy in the future. To facilitate future clinical application, it needs to be further developed and clinically validated using EAM techniques such as FIRM or ECGI, which allow for identifying patient-specific RD locations. Indeed, these tools have been used by previous computational studies investigating RD locations by Boyle et al. [19,20] to validate their findings and show a fair correlation between model predictions and clinical findings. Moreover, our workflow can be extended to incorporate further patient-specific details, such as atrial fibre orientation and electrophysiological heterogeneity. Such details have been shown to play important roles in the genesis of AF [23,24,49], and their integration may substantially increase the predictive power of the models.

### Limitations

Previous computational modelling studies have reported the dynamics of RDs to be dependent on the methodology for modelling fibrosis [43]. We adopted an approach where atrial models relied on information obtained from patient MR imaging. Our study did not consider the influence of other patient-specific factors such as atrial anisotropy and electrophysiological heterogeneity, which may contribute to drift of the RDs observed in the realistic LA geometries [50]. Atrial fibre orientation is known to be complex [51] and can also have significant effects on atrial conduction [23,50]. However, fibre orientation was not incorporated in this study due to the absence of patience-specific data in this regard. Future studies will aim to incorporate information about fibre orientation into patient-specific atrial models based on recently proposed rule-based approaches [52]. The modelling approach presented in the current study is entirely based on patient imaging-derived data available in the clinic.

Note that, although we have used a simple and phenomenological aFK model for the LA simulations, our results are consistent with other studies [14,15,53] performed using more detailed atrial myocyte model such as Coutemanche-Ramirez-Nattel (CRN) model [54], which have also reported anchoring of RDs to fibrotic regions. Moreover, we tested our protocol on 3D slab with the CRN atrial cell model and demonstrated similar behaviour of the RDs (further details provided in the S4 Fig).

In this study we have only considered fully transmural fibrosis. However, experimental recordings by Verheule et al. [55,56], have demonstrated the existence of endomysial fibrosis which develops exclusively within the epicardial layer and accompanies the transition from persistent to permanent AF in goats. Moreover, a recent computational study by Gharaviri et al. [57] in a human atrial model has proven that such fibrotic patterns could result in increased breakthroughs and endo-epicardial dissociations. In future studies, intramural fibrosis should be incorporated into atrial models to analyse its effects on the RD dynamics and CA strategy. However, the non-invasive imaging of such fibrotic regions is limited by current resolution of LGE-MRI technology, which is comparable to the transmural distance in thin atrial walls.

In the virtual ablation study, all the CA lesions were applied simultaneously. However, in the clinic they are applied in a sequential manner. This could potentially influence the outcome of virtual ablation. Furthermore, the virtual lesions were ‘perfect lesions’ that were fully transmural and maintain a complete conduction block. This is hard to achieve clinically and depends on the operator and the location of ablation. Another limitation of this study is that our AF simulation workflow used the cross-filed protocol rather than fast pacing (e.g., by McDowell et al. [14]) to initiate AF. Although the workflow is designed to predict the ultimate RD anchoring sites rather than the initiation sites, the latter may also be important for AF termination and its recurrence prevention. However, our workflow could be easily adjusted to include fast pacing for the evaluation of both RD anchoring and initiation mechanisms.

## Conclusion

Patient-specific LA model simulations showed that RD sustaining AF typically anchored to large fibrotic patches or their BZ, with specific pattern of the RD movement through/around a patch influenced by the size and shape of the patch. Therefore, typical RD locations were determined by unique patient-specific distributions of fibrotic tissue, identifying areas that may potentially be targeted by therapy. These results may be particularly relevant to AF patients with high fibrosis burden in the LA, where the model-predicted TAs could inform CA. Performing virtual ablations on the TAs and connecting them to the nearest PVs or MV has superior anti-fibrillatory effect compared to ablating the TAs alone, as well as compared to clinically accepted strategies such as PVI.

## Supporting information

S1 Text Supplementary Material, including Courtemanche-Ramirez-Nattel (CRN) model. (PDF)

S1 Table The mean frequencies (MF) calculated before and after virtual CA in Patient 2 (bottom) and 3 (top).
(PDF)

S1 Fig The pipeline for identifying TAs from the RD probability map. Sim: simulation.
(TIF)

S2 Fig Identifying RD location maps from patient-specific tip probability maps. (A) Shows the tip probability map across the entire LA model of patient P1 and (B) shows the locations of target areas identified by thresholding the normalised probability map (A) at two levels (yellow, Th1: 0.2) and (orange, Th2: 0.15) and overlaid on the fibrosis map (greyscale).
(TIF)

S3 Fig Patient-specific RD location maps–catheter ablation targets. The images show colour-coded fibrosis distributions (greyscale) in the 6 patient-specific LA models, with the TAs (with fibrosis: yellow and without fibrosis: orange) superimposed. In Utah 4 patient (P1), TAs are seen at specific locations within fibrotic patches. In Utah 3 patients (P2, P3 and P4), TAs are distributed at the BZ between fibrotic patches and healthy tissue. In Utah 2 patients (P5 and P6), TAs are seen mostly on the LA wall with some near small patches.
(TIF)

S4 Fig Anchoring of RDs to fibrotic patches with CRN atrial cell model. (A) The voltage map for RD is shown with positions of initiation marked as (1) and (2). The tip trajectories of the RDs initiated from these positions are shown in (B) and (C). The target areas computed for this scenario is shown in panel (D), marked in yellow and fibrotic patch in black.
(TIF)
