# Supplementary material for: Identifying locations of re-entrant drivers from patient-specific distribution of fibrosis in the left atrium
Source: PLoS Comput Biol. 2020 Sep 23;16(9):e1008086. doi: 10.1371/journal.pcbi.1008086 (PMC7535127; doi:10.1371/journal.pcbi.1008086)
Supplement: S1 Text — (PDF) [file pcbi.1008086.s001.pdf]

Supplementary Material

Identifying Locations of Re-entrant Drivers from Patient-Specific Distribution of Fibrosis in the Left Atrium

Aditi Roy[1], Marta Varela[1], Henry Chubb[2], Robert MacLeod[3], Jules C. Hancox[4], Tobias Schaeffter[5], Oleg Aslanidi*[1]

[1]*Department of Biomedical Engineering, School of Biomedical Engineering & Imaging Sciences, King's College London, St Thomas' Hospital, London, SE1 7EH, UK*; [2]*Cardiothoracic Surgery, Stanford University, USA;* [3]*Bioengineering Department, University of Utah, Salt Lake City, Utah;* [4]*School of Physiology and Pharmacology, Cardiovascular Research Laboratories, University of Bristol, Bristol, United Kingdom;* [5]*Physikalisch-Technische Bundesanstalt, Abbestr. 2-12, 10587 Berlin, Germany;*

*** Correspondence: Oleg Aslanidi**

School of Biomedical Engineering & Imaging Sciences

King's College London

London SE1 7EH, United Kingdom

oleg.aslanidi@kcl.ac.uk; Phone: +44(0)2071887188

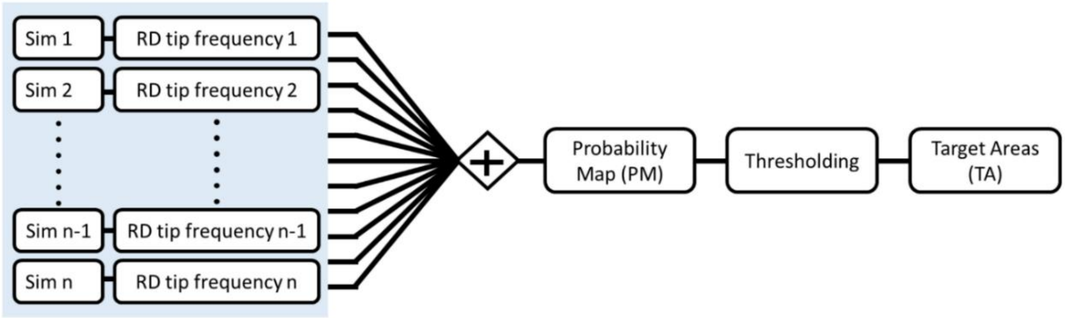

S1 Fig: The pipeline for identifying TAs from the RD probability map. Sim: simulation

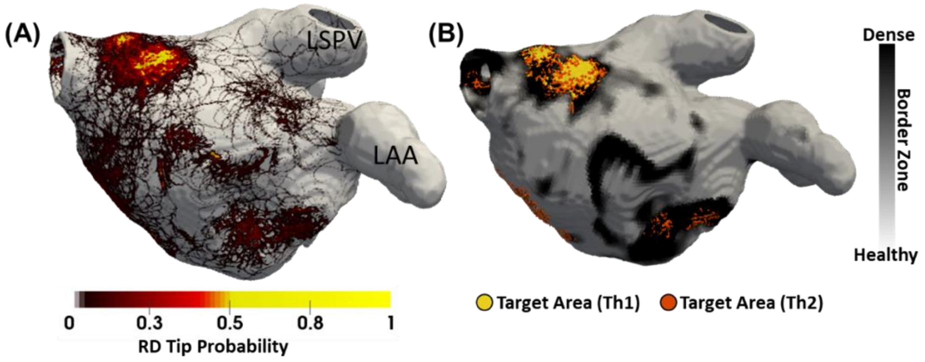

S2 Fig: Identifying RD location maps from patient-specific tip probability maps. (A) Shows the tip probability map across the entire LA model of patient P1 and (B) shows the locations of target areas identified by thresholding the normalised probability map (A) at two levels (yellow, Th1: 0.2) and (orange, Th2: 0.15) and overlaid on the fibrosis map (greyscale).

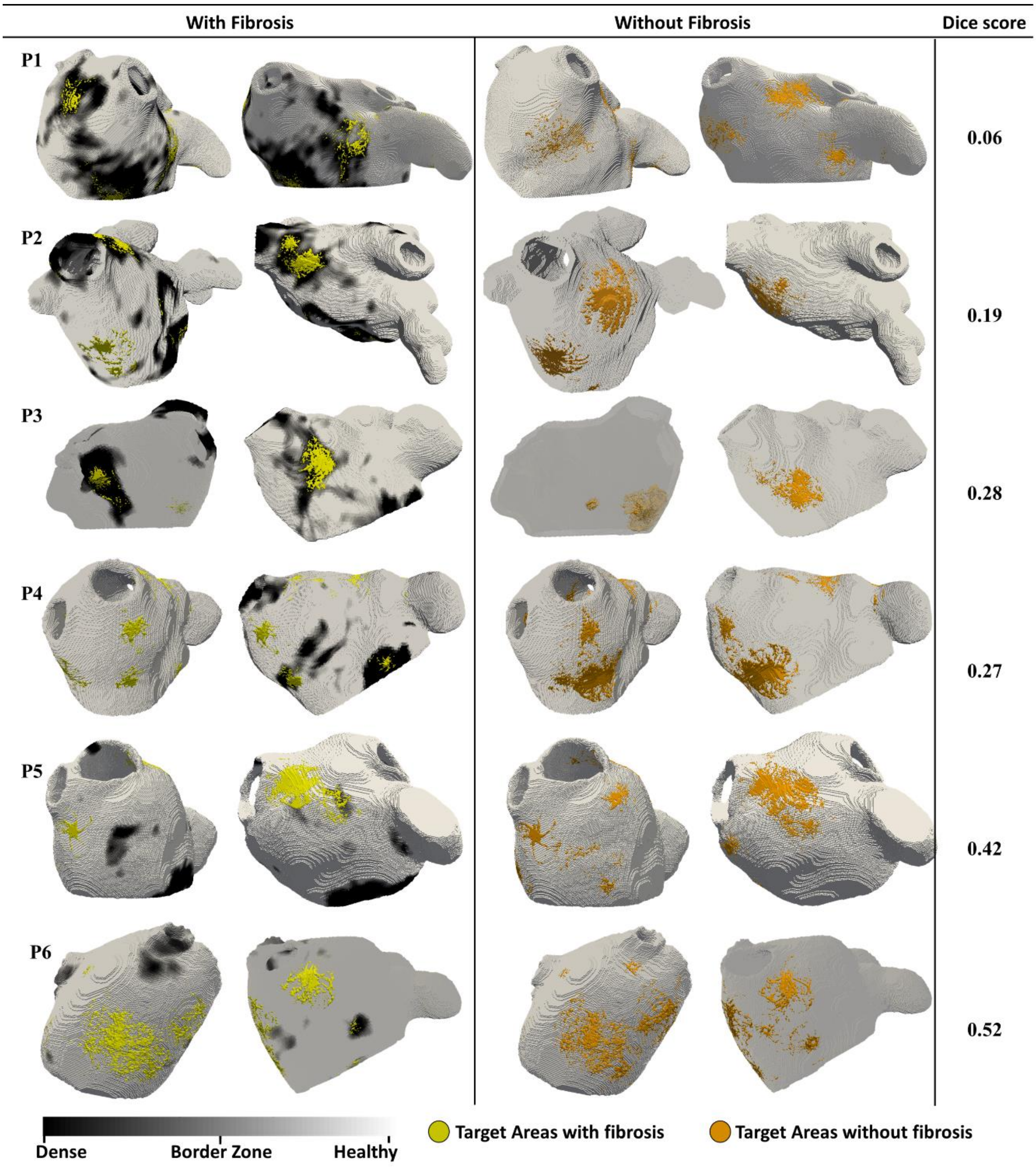

S3 Fig: Patient-specific RD location maps – catheter ablation targets. The images show colour-coded fibrosis distributions (greyscale) in the 6 patient-specific LA models, with the TAs (with fibrosis: yellow and without fibrosis: orange) superimposed. In Utah 4 patient (P1), TAs are seen at specific locations within fibrotic patches. In Utah 3 patients (P2, P3 and P4), TAs are distributed at the BZ between fibrotic patches and healthy tissue. In Utah 2 patients (P5 and P6), TAs are seen mostly on the LA wall with some near small patches.

AF Cases	Control	TA	TA+L	PVI+TA+L
**1**	10.06 ± 0.19	6.25 ± 0.02	5.62 ± 0.01	4.14 ± 0.30
**2**	7.90 ± 0.30	8.11 ± 0.09	8.11 ± 0.09	0
**3**	10.06 ± 0.20	9.37 ± 0.03	9.37 ± 0.03	0
**4**	9.87 ± 0.68	5.84 ± 0.30	5.62 ± 0.00	4.09 ± 0.31
**5**	10.23 ± 0.32	9.37 ± 0.03	9.37 ± 0.03	4.12 ± 0.31
**6**	10.21 ± 0.34	5.94 ± 0.31	4.36 ± 0.11	4.03 ± 0.31
**7**	10.19 ± 0.32	5.87 ± 0.31	4.35 ± 0.13	4.04 ± 0.31
**8**	10.10 ± 0.28	10.09 ± 0.25	10.08 ± 0.25	4.03 ± 0.31

Patient 3

AF Cases	Control	TA	TA+L	PVI+TA+L
**1**	10.03 ± 0.26	10.03 ± 0.26	10.03 ± 0.27	0
**2**	10.07 ± 0.26	7.50 ± 0.11	0	0
**3**	10.10 ± 0.28	10.10 ± 0.28	10.10 ± 0.28	4.64 ± 1.45
**4**	10.12 ± 0.27	7.50 ± 0.09	0	0
**5**	10.41 ± 0.31	6.22 ± 0.15	0	0
**6**	10.10 ± 0.29	10.10 ± 0.29	10.10 ± 0.29	4.38 ± 0.00
**7**	10.40 ± 0.34	10.14 ± 0.34	0	0
**8**	10.43 ± 0.29	6.25 ± 0.07	0	0
**9**	10.02 ± 0.21	10.02 ± 0.21	10.02 ± 0.21	10.02 ± 0.21
**10**	9.62 ± 0.62	8.12 ± 0.10	0	0

Patient 2

S1 Table: The mean frequencies (MF) calculated before and after virtual CA in Patient 2 (bottom) and 3 (top).

Courtemanche-Ramirez-Nattel (CRN) model

In order to check independence of the simulation results on the choice of model, we performed on a 3D slab using the Courtemanche-Ramirez-Nattel (CRN) model [1], which describes atrial myocyte in more detail. The latter has also been modified to match the restitution properties of remodeled atrial cells [2]. The diffusion coefficient for the CRN model was chosen as 0.16 $mm^2 ms^{-1}$ to produce the atrial conduction velocity of 0.60 $m s^{-1}$, same as that simulated with the FK model. The spatial step of 0.3 mm and temporal step of 0.005 ms were used for modified CRN model, for which numerical stability has been shown previously [3]. The trajectory of the RDs tip was tracked for 6s s in each simulation.

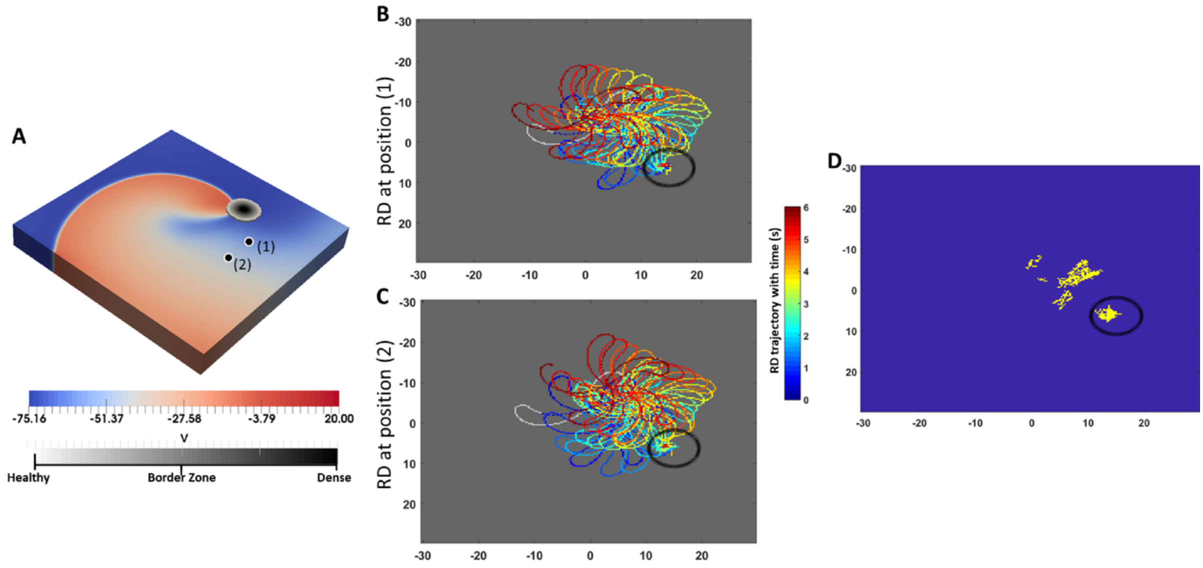

S4 Fig: Anchoring of RDs to fibrotic patches with CRN atrial cell model. (A) The voltage map for RD is shown with positions of initiation marked as (1) and (2). The tip trajectories of the RDs initiated from these positions are shown in (B) and (C). The target areas computed for this scenario is shown in panel (D), marked in yellow and fibrotic patch in black.

Simulations of the 3D slab with the CRN atrial cell model demonstrated similar behaviour of the RDs (further details provided in the supplementary Figure S4). The RD tip in the CRN-based model also meandered to form a flower-like pattern. Similar to the aFK-based model, anchored to the fibrotic patch, indicating that the anchoring phenomena was model-

independent. Our results are consistent with other studies [4–6] performed using CRN model, which have also reported anchoring of RDs to fibrotic regions. These simulations were performed on a 3D slab as it is extremely computationally expensive to run the full set of simulations on patient-specific LA models with a higher order model

References

1. Courtemanche M, Ramirez RJ, Nattel S. Ionic mechanisms underlying human atrial action potential properties: insights from a mathematical model. Am J Physiol. 1998 Jul;275(1 Pt 2):H301-21.
2. Colman MA, Aslanidi O, Kharche S, Boyett MR, Garratt C, Hancox JC, et al. Pro-arrhythmogenic effects of atrial fibrillation-induced electrical remodelling: Insights from the three-dimensional virtual human atria. J Physiol. 2013 Sep 1;591(17):4249–72.
3. Aslanidi O, Colman MA, Stott J, Dobrzynski H, Boyett MR, Holden A V., et al. 3D virtual human atria: A computational platform for studying clinical atrial fibrillation. Prog Biophys Mol Biol. 2011 Oct;107(1):156–68.
4. McDowell KS, Zahid S, Vadakkumpadan F, Blauer J, MacLeod RS, Trayanova N. Virtual electrophysiological study of atrial fibrillation in fibrotic remodeling. PLoS One. 2015 Feb 18;10(2):e0117110.
5. Zahid S, Cochet H, Boyle PM, Schwarz EL, Whyte KN, Vigmond EJ, et al. Patient-derived models link re-entrant driver localization in atrial fibrillation to fibrosis spatial pattern. Cardiovasc Res. 2016 Jun 1;110(3):443–54.
6. Boyle PM, Zghaib T, Zahid S, Ali RL, Deng D, Franceschi WH, et al. Computationally guided personalized targeted ablation of persistent atrial fibrillation. Nat Biomed Eng. 2019;3(11):870–9.
