# Supplementary material for: Identifying locations of re-entrant drivers from patient-specific distribution of fibrosis in the left atrium
Source: PLoS Comput Biol. 2020 Sep 23;16(9):e1008086. doi: 10.1371/journal.pcbi.1008086 (PMC7535127; doi:10.1371/journal.pcbi.1008086)
Supplement: S1 Table — The mean frequencies (MF) calculated before and after virtual CA in Patient 2 (bottom) and 3 (top). (PDF) [file pcbi.1008086.s002.pdf]

Patient 3

AF Cases	Control	TA	TA+L	PVI+TA+L
**1**	10.06 ± 0.19	6.25 ± 0.02	5.62 ± 0.01	4.14 ± 0.30
**2**	7.90 ± 0.30	8.11 ± 0.09	8.11 ± 0.09	0
**3**	10.06 ± 0.20	9.37 ± 0.03	9.37 ± 0.03	0
**4**	9.87 ± 0.68	5.84 ± 0.30	5.62 ± 0.00	4.09 ± 0.31
**5**	10.23 ± 0.32	9.37 ± 0.03	9.37 ± 0.03	4.12 ± 0.31
**6**	10.21 ± 0.34	5.94 ± 0.31	4.36 ± 0.11	4.03 ± 0.31
**7**	10.19 ± 0.32	5.87 ± 0.31	4.35 ± 0.13	4.04 ± 0.31
**8**	10.10 ± 0.28	10.09 ± 0.25	10.08 ± 0.25	4.03 ± 0.31

Patient 2

AF Cases	Control	TA	TA+L	PVI+TA+L
**1**	10.03 ± 0.26	10.03 ± 0.26	10.03 ± 0.27	0
**2**	10.07 ± 0.26	7.50 ± 0.11	0	0
**3**	10.10 ± 0.28	10.10 ± 0.28	10.10 ± 0.28	4.64 ± 1.45
**4**	10.12 ± 0.27	7.50 ± 0.09	0	0
**5**	10.41 ± 0.31	6.22 ± 0.15	0	0
**6**	10.10 ± 0.29	10.10 ± 0.29	10.10 ± 0.29	4.38 ± 0.00
**7**	10.40 ± 0.34	10.14 ± 0.34	0	0
**8**	10.43 ± 0.29	6.25 ± 0.07	0	0
**9**	10.02 ± 0.21	10.02 ± 0.21	10.02 ± 0.21	10.02 ± 0.21
**10**	9.62 ± 0.62	8.12 ± 0.10	0	0

S1 Table: The mean frequencies (MF) calculated before and after virtual CA in Patient 2 (bottom) and 3 (top).
