# Supplementary figures and images for: Identifying locations of re-entrant drivers from patient-specific distribution of fibrosis in the left atrium
Source: PLoS Comput Biol. 2020 Sep 23;16(9):e1008086. doi: 10.1371/journal.pcbi.1008086 (PMC7535127; doi:10.1371/journal.pcbi.1008086)

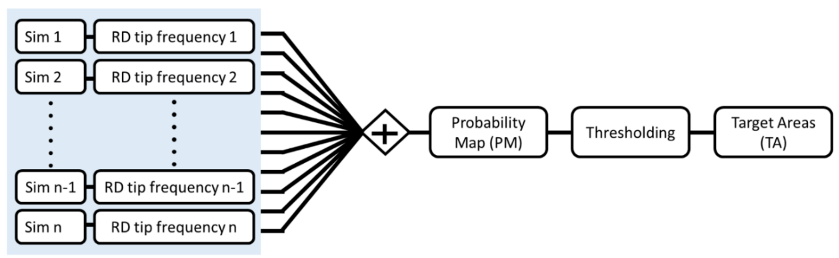

Supplement: S1 Fig — Sim: simulation. (TIF) [file pcbi.1008086.s003.tif]

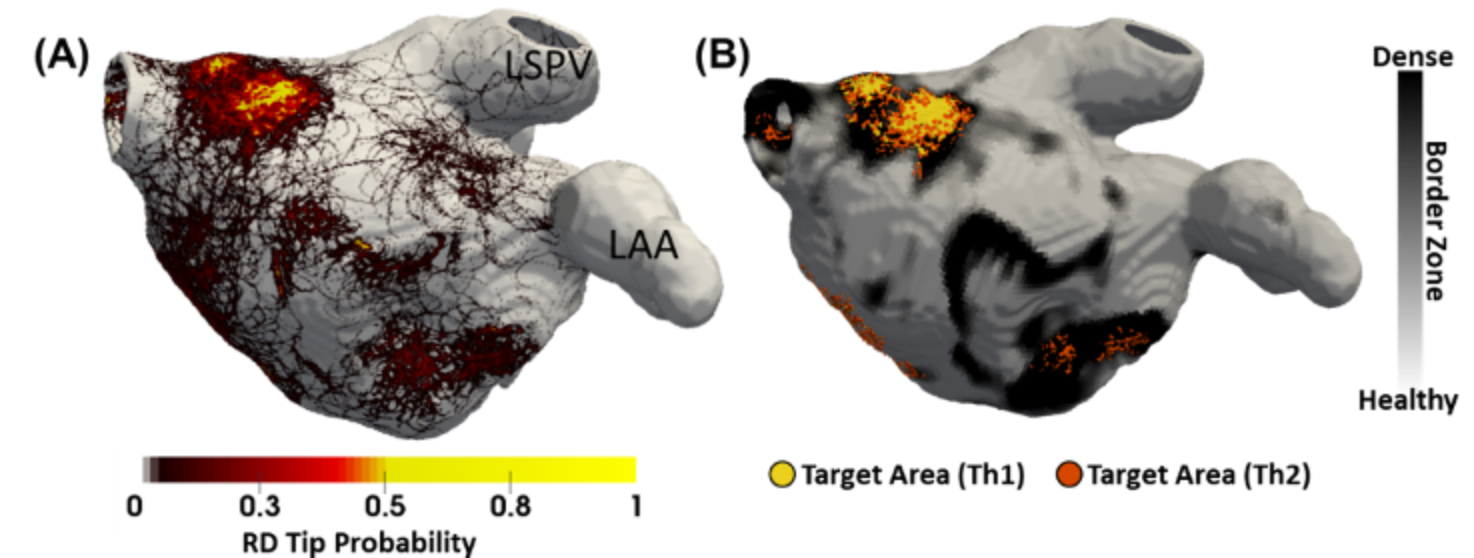

Supplement: S2 Fig — (A) Shows the tip probability map across the entire LA model of patient P1 and (B) shows the locations of target areas identified by thresholding the normalised probability map (A) at two levels (yellow, Th1: 0.2) and (orange, Th2: 0.15) and overlaid on the fibrosis map (greyscale). (TIF) [file pcbi.1008086.s004.tif]

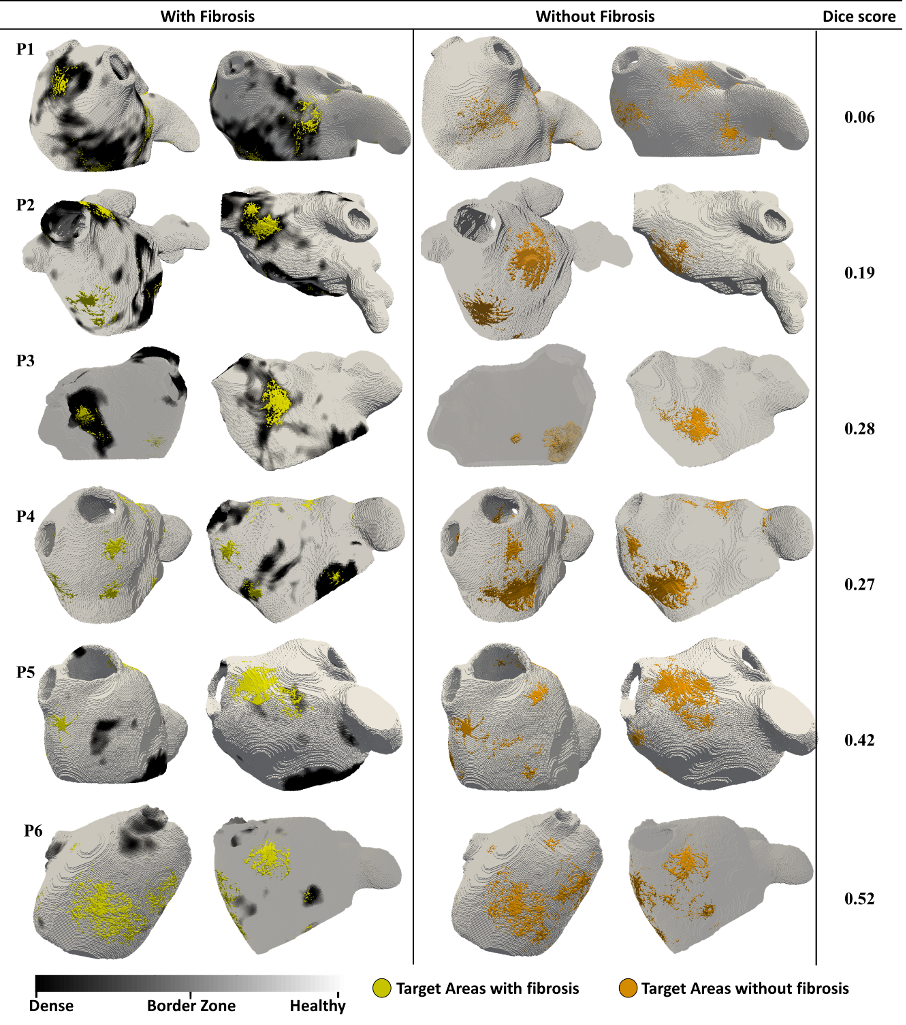

Supplement: S3 Fig — The images show colour-coded fibrosis distributions (greyscale) in the 6 patient-specific LA models, with the TAs (with fibrosis: yellow and without fibrosis: orange) superimposed. In Utah 4 patient (P1), TAs are seen at specific locations within fibrotic patches. In Utah 3 patients (P2, P3 and P4), TAs are distributed at the BZ between fibrotic patches and healthy tissue. In Utah 2 patients (P5 and P6), TAs are seen mostly on the LA wall with some near small patches. (TIF) [file pcbi.1008086.s005.tif]

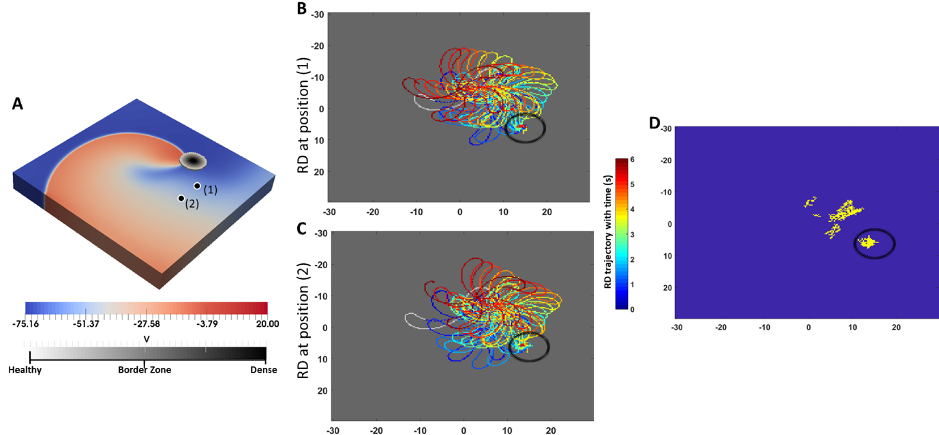

Supplement: S4 Fig — (A) The voltage map for RD is shown with positions of initiation marked as (1) and (2). The tip trajectories of the RDs initiated from these positions are shown in (B) and (C). The target areas computed for this scenario is shown in panel (D), marked in yellow and fibrotic patch in black. (TIF) [file pcbi.1008086.s006.tif]
